# Imbalanced Expression of *Vcan* mRNA Splice Form Proteins Alters Heart Morphology and Cellular Protein Profiles

**DOI:** 10.1371/journal.pone.0089133

**Published:** 2014-02-20

**Authors:** Tara A. Burns, Maria T. Dours-Zimmermann, Dieter R. Zimmermann, Edward L. Krug, Susana Comte-Walters, Leticia Reyes, Monica A. Davis, Kevin L. Schey, John H. Schwacke, Christine B. Kern, Corey H. Mjaatvedt

**Affiliations:** 1 Departments of Regenerative Medicine & Cell Biology, Medical University of South Carolina, Charleston, South Carolina, United States of America; 2 Department of Cell and Molecular Pharmacology, Medical University of South Carolina, Charleston, South Carolina, United States of America; 3 Institute of Surgical Pathology, University Hospital Zurich, Zurich, Switzerland; 4 Department of Biochemistry, Vanderbilt University School of Medicine, Nashville, Tennessee, United States of America; Heart Science Centre, Imperial College London, United Kingdom

## Abstract

The fundamental importance of the proteoglycan versican to early heart formation was clearly demonstrated by the *Vcan* null mouse called heart defect (*hdf*). Total absence of the *Vcan* gene halts heart development at a stage prior to the heart’s pulmonary/aortic outlet segment growth. This creates a problem for determining the significance of versican’s expression in the forming valve precursors and vascular wall of the pulmonary and aortic roots. This study presents data from a mouse model, *Vcan* ^(tm1Zim)^, of heart defects that results from deletion of exon 7 in the *Vcan* gene. Loss of exon 7 prevents expression of two of the four alternative splice forms of the *Vcan* gene. Mice homozygous for the exon 7 deletion survive into adulthood, however, the inability to express the V2 or V0 forms of versican results in ventricular septal defects, smaller cushions/valve leaflets with diminished myocardialization and altered pulmonary and aortic outflow tracts. We correlate these phenotypic findings with a large-scale differential protein expression profiling to identify compensatory alterations in cardiac protein expression at E13.5 post coitus that result from the absence of *Vcan* exon 7. The *Vcan* ^(tm1Zim)^ hearts show significant changes in the relative abundance of several cytoskeletal and muscle contraction proteins including some previously associated with heart disease. These alterations define a protein fingerprint that provides insight to the observed deficiencies in pre-valvular/septal cushion mesenchyme and the stability of the myocardial phenotype required for alignment of the outflow tract with the heart ventricles.

## Introduction

During embryonic development, the extracellular matrix plays a central role in restructuring the single heart tube into a mature multi-chambered organ. To date, only two structural components of the extracellular matrix, versican and its binding partner hyaluronan, have been shown to be indispensably required for successful completion of this morphogenetic transition. Complete loss of either of these matrix molecules results in early embryonic lethality with a failure of endocardial cushion formation and a highly dilated myocardium of the primitive heart tube [1], [2].

**Figure 1:**
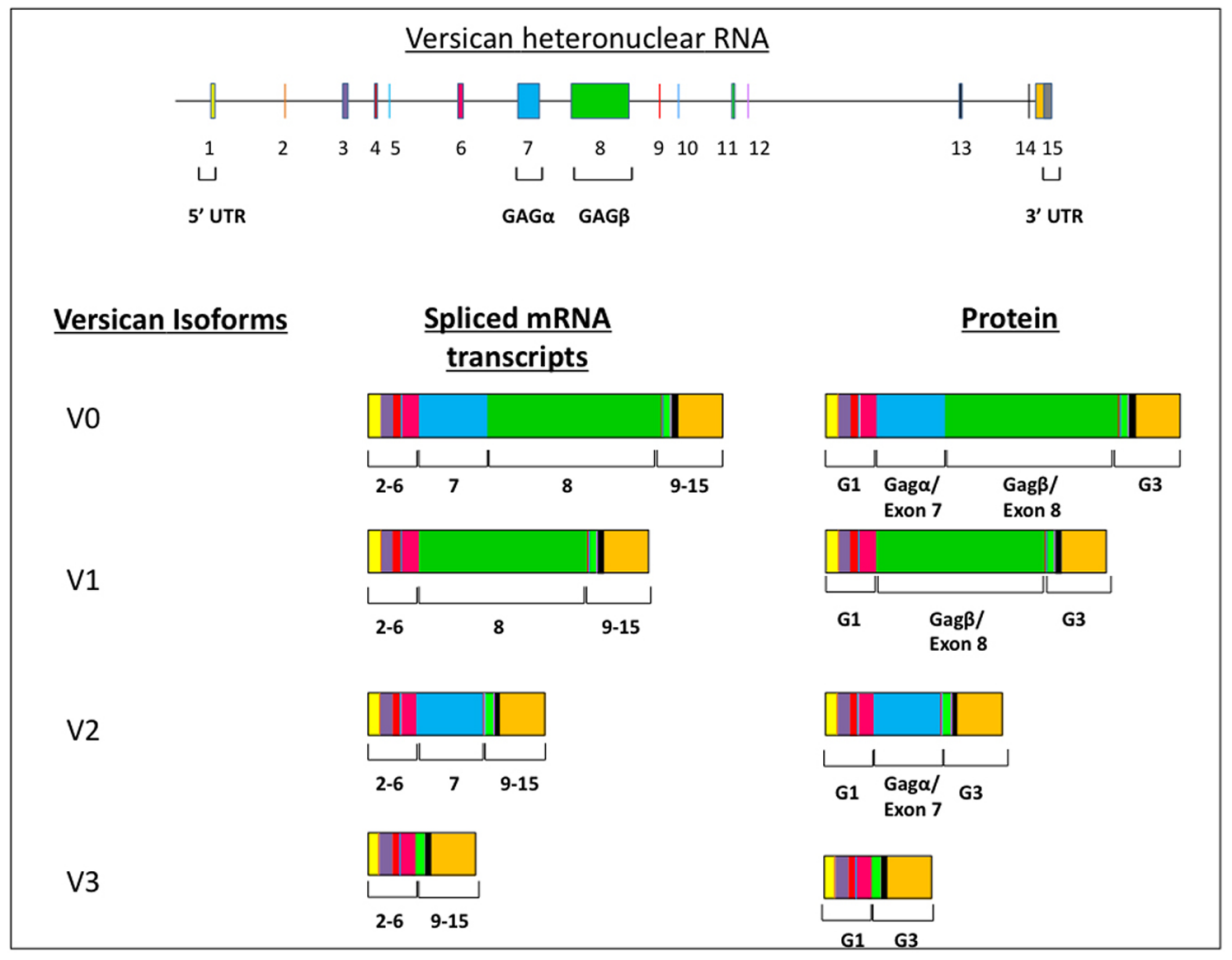
Splice forms of versican. The *Vcan* gene consists of 15 exons. Four mRNA and corresponding protein variants of the core protein (V0, V1, V2, V3) are derived from alternative splicing of exons 7 (blue boxes) and/or 8 (green boxes) into mRNA of *Vcan* gene. The glycosaminoglycan attachment domains GAG α & β are encoded by exon 7 (blue) and 8 (green) respectively. Deletion of exon 7 (blue) results in the loss of both the V2 and V0 variants. Exons 2–6 comprise the G1 domain that binds hyaluronan and 9–15 the G3 domain that also can interact with other ECM molecules.

Versican is a chondroitin sulfate proteoglycan that was first identified in fibroblastic extracts [3]. Versican is abundantly expressed within the extracellular matrix compartment of the developing and mature cardiovascular system. Alterations in its expression have been associated with vascular disease [4] and its expression is required early for normal early heart development [5]. The versican gene (*Vcan*; formerly *Cspg2*) consists of 15 exons and spans a region of approximately 100 kb [6], [7]. The exons encode modular protein domains whose presence in the protein core is regulated by alternative splicing of the mRNA [8], [9]. At least 4 different mRNA splice forms (V0, V1, V2 and V3) have been identified in adult tissue and are produced by the alternative splicing of exons 7 and 8 (Fig. 1). Several studies of expression have shown that these alternatively spliced mRNA forms are differentially expressed in specific tissues types, e.g. brain, smooth muscle, tumors, suggesting that the regulation of functional domains within versican are required for differentiation and persistence of a tissue’s phenotype [9]–[15]. Additionally, the activity of versican’s protein domains may be altered by selective proteolysis. Two of the splice forms V0 & V1 have specific cleavage sites (DPEAAE) found within target substrates of metalloproteinase ADAMTS-1 [16]. Proteolytic processing of versican occurs during changes in the vascular system [16]–[18] and during critical stages of heart development [19]–[21].

As shown by the *heart defect* mouse (*hdf*), the absence of versican protein results in early embryonic lethality [5]. Embryonic mice homozygous for the *hdf* allele display severe cardiac defects including absence of pre-valvular endocardial cushions, loss of anterior (second) heart field structures and a thin, dilated myocardium. Similarly, loss of a subdomain of the G1 domain (found in all *Vcan* splice forms) results in embryonic lethality of homozygous embryos on a congenic and mixed background. However, on the mixed background some embryos survive longer and have ventricular septal defects [22]. The purpose of the this study was to determine the functional significance of an imbalance in *Vcan*’s alternative splice forms at later stages of heart development using a versican alternative splice form deficient mouse model. The *Vcan* ^(tm1Zim)^ mouse, expresses only the V1 and V3 splice forms of versican in the heart and survive embryogenesis [23]. We demonstrate that mice homozygous for the *Vcan* ^(tm1Zim)^ deletion have less severe cardiac defects than the *hdf* homozygous mice. Differential protein expression profiling using isobaric tag reagents for absolute and relative quantitation (iTRAQ) [24] coupled with shotgun proteomics was used to identify some of the responsive changes in cellular protein expression that result from the loss of versican V2/V0 splice forms.

## Materials and Methods

### Ethics Statement

This study was carried out in strict accordance with the recommendations in the Guide for the Care and Use of Laboratory Animals of the National Institutes of Health. The protocol (AR#1572) was approved by the Institutional Animal Care & Use Committee (IACUC) at the Medical University of South Carolina. Wild-type C57BL6/J and *Vcan^(tm1Zim)^* hearts were collected from timed pregnant dams and staged according to Theiler [25] before being processed for analysis as described below.

### Generation of the *Vcan* ^(tm1Zim)^ Mice

The *Vcan* ^(tm1Zim)^ mice were created and characterized as described elsewhere [23] Briefly, by homologous recombination, an endoplasmic reticulum retention signal, a stop codon and a floxed neo-tk cassette were incorporated in exon 7 of the versican gene (V0/V2 neo-tk). The selection cassette was removed by crossing these heterozygous mice with a CRE-deleter strain eliminating exon 7 [26], [27]. Genotyping of genomic DNA from progeny of hemizygous matings was performed by PCR using the following exon 7 specific primers: 5′-GAGAGGACAGAAACACCAAG-3′, 5′-ACTGTGGGTCAAATGAACTC-3′ designed to yield a 454 bp product from the mutated allele and a 312 bp product from the wild-type allele. The absence of the V0 and V2 splice forms was independently confirmed in our laboratory by immunohistochemistry and western blot analysis.

### Rapid Confocal Morphological Analysis of Intact Embryonic Mouse Hearts

To evaluate the whole mouse embryo hearts by optical sectioning, we adapted a confocal fluorescence imaging technique originally used with avian hearts [28]. Embryonic day 13.5 hearts were perfused in situ with modified saline (buffered saline solution containing 30 mM KCl & 10 ug/ml verapamil) to remove blood and suspend all the hearts in the same state of contraction. Buffered saline containing FITC conjugated poly-L-lysine was then perfused into the hearts to enhance visualization of the endothelial lining of the hearts and vessels prior to fixation with 4% paraformaldehyde solution. Whole heart samples were then mounted in precise orientation, processed and cleared for multi-channel confocal optical sectioning (Leica TCS SP2 SOBS confocal microscope).

### 3D Collagen Gel Endothelial-mesenchymal (EMT) Assay

Embryos (E10.5 pc) were dissected from the mouse decidua and extra embryonic tissues and pooled into Earle’s balanced salt solution (Gibco). Hearts were dissected from the embryos and the AV regions were removed, and cut to expose the lumen. The AV explants were placed immediately onto the surface of drained collagen gels with 1 explant per well, and incubated at 37°C for 3–4 hr to allow attachment of the explants onto the collagen gel surface as previously described [29], [30]. A total of 0.5 ml of DMEM (5% fetal bovine serum, antibiotics) was added to each well and incubated for a total of 48 hr at 37°C. The number of mesenchymal cells was assessed by observing the living cultures with a Zeiss Axiovert 100 inverted microscope with Hoffman phase contrast optics and “optically sectioning” the collagen gel by changing focal planes to reveal the presence of any cells beneath the surface of the endothelial monolayer for counting. Total mesenchymal cell counts were made from each explant and then later grouped according to genotype as determined in separate PCR assays performed after the explant EMT experiments were scored. Statistical analysis was performed to determine if significant differences exist between the mean values obtained from wild-type and mutant using the unpaired (student’s) t-test within the Prism GraphPad analysis software.

### Histology and Immunofluorescence

Hearts dissected from postnatal mice or embryos were fixed in 4% paraformaldehyde, embedded in paraffin, and sectioned as previously described [19]. For immunohistochemistry, deparaffinized sections were rehydrated through a graded series of ethanol solutions to phosphate buffered saline (PBS). Hematoxylin and eosin staining followed standard procedures in the lab. Sections used for immunostaining were pretreated to unmask any cryptic antigens using a high-temperature citric acid solution according to directions (H-3300, Vector Laboratories, Burlingame, CA). Sections were blocked 1 hr at room temperature with blocking buffer (PBS, Sigma containing 3% normal goat serum (NGS, Cappel, Malvern, PA), and 1% bovine serum albumin (catalog no. B4287, Sigma) then incubated overnight at 4°C with primary antibody diluted in blocking buffer (anti- GAG-α and -β; Chemicon, Temecula, CA were diluted to 5 µg/ml; periostin antibody was diluted to 5 µg/ml. After primary antibody incubations, specimens were washed 5 times in PBS and incubated at room temperature with fluorochrome-conjugated secondary antibody (Jackson Immunoresearch, West Grove, PA) diluted in blocking buffer. All samples were washed extensively in PBS, nuclei were labeled with 1 µg/ml propidium iodide (Molecular Probes C-7590) in PBS for 5 min prior to the final washes in PBS and slides were cover-slipped using DABCO mounting media (Sigma). Controls included the use of the secondary antibody only and use of pre-immune serum. Immunostained sections were analyzed using a Leica TCS SP2 AOBS Confocal Microscope System (Leica Microsystems,Inc., Exton, PA). For 3-dimensinal reconstruction and morphometric analysis micrographs of 7 µm thick serial sections through E13.5 pc hearts were stacked and aligned within the AMIRA (v5.4) 3D reconstruction software as previously described [31], [32]. Total volume measurements were made of the selected structures using the Materials/Statistics tool for each of the reconstructed hearts (n = 3 for each genotype) and statistical analysis performed to compare significant differences between the mean values by unpaired (student’s) t-test using the Prism GraphPad analysis software.

### iTRAQ Analysis

Pregnant females at E13.5 post coitus (pc) were anesthetized and euthanized following approved protocols of the Division of Laboratory Animal Resources at MUSC and AAALAC guidelines. Hearts of homozygous *Vcan* ^(tm1Zim)^ mutant and wild-type E13.5 p.c. embryos obtained from multiple litters were removed by micro-dissection, bisected and rinsed with PBS, frozen in liquid nitrogen and then finely crushed in a liquid nitrogen cooled stainless steel mortar and pestle (Model 59012N; BioSpec Products, Barlesville, OK). Protein profiling was performed on the pooled hearts of 5 animals from each group in order to obtain sufficient protein for analysis. The samples were homogenized (1∶10, w/v) in ice cold 20 mM HEPES, pH 8/4°C, containing 1 M urea in a 1 mL ground glass tissue grinder then subjected to 770×g for 10 min at 4°C to remove nuclei and undisassociated tissue. The resulting supernatants were subjected to centrifugation at 150,000×g for 90 min at 4°C (Beckman Optima TLX ultracentrifuge with TKS-55 rotor) to obtain a combined urea soluble and insoluble fraction. The protein concentration in the urea soluble fraction was determined by the BCS method (Pierce, Rockville, IL) using bovine serum albumin as standard. The samples were reduced and alkylated prior to digestion with LysC and trypsin at a ratio of 1∶10 [33].

Heart preparations from the Wt (wild type), and *Vcan* ^(tm1Zim)^ mutant E13.5 pc hearts (n = 5 per group) were labeled with the “isobaric tag reagents for absolute and relative quantitation” (iTRAQ™) (Applied Biosystems- Framingham, MA). Duplicate samples (90 µg of protein each) from wild-type and *Vcan* ^(tm1Zim)^ mutant hearts were each labeled with one of the 4 unique isobaric tags (114-wt sample 1; 116-wt sample 2; 115- *Vcan* ^(tm1Zim)^ sample 1; 117- *Vcan* ^(tm1Zim)^ sample 2) and all run in parallel through the iTRAQ analysis. This strategy created an internal control for the analysis since the ratio for any one detected protein when compared to the value for the same protein in the duplicate sample should equal 1 (114-wt/116-wt and 115-*Vcan* ^(tm1Zim)^/117-*Vcan* ^(tm1Zim)^). The combined samples were subjected to cationic exchange/reversed-phase HPLC fractionation and mass spectrometry analysis for protein identification and quantification. Protein quantification for all of the iTRAQ peptides found was calculated using a GPS software and the statistical model for iTRAQ proteomic analysis [34], [35]. The magnitude and significance of change in protein expression between the two experimental groups was analyzed using a software package named iQuantitator that is designed specifically for iTRAQ [35]. The software has been used in other studies of similar design to ours to identify relevant proteins for further study from multiple pooled samples grouped within a single iTRAQ analysis [36]–[38]. The software infers the genotype-dependent changes in expression using Bayesian statistical methods. Inference is based on a well-known statistical model and uses Markov-Chain Monte Carlo methods (Gibbs Sampling). We generated the means, medians, and 95% credible intervals (upper and lower) for each genotype-dependent change in protein expression by using peptide-level data for each component peptide, and integrating the data across the control and experimental group of pooled samples. For proteins whose iTRAQ ratios were down regulated in the pooled null hearts, the extent of down-regulation was considered further if the null value of 1 was above the upper limit of the credible interval. Conversely, for proteins whose iTRAQ ratios were up-regulated in null hearts, the extent of up-regulation was considered further if the lower limit of the credible interval had a value greater than 1. The width of these credible intervals depends on the data available for a given protein. Since the number of peptides observed and the number of spectra used to quantify the change in expression for a given protein are taken into consideration, it is possible to detect small but significant changes in up- or down-regulation when many peptides are available.

### Western Blot Analysis

Hearts of homozygous *Vcan* ^(tm1Zim)^ mutant and wild-type E13.5 p.c. embryos were obtained by microdissection and proteins extracted as described for iTRAQ analysis. The protein concentration in the urea soluble fraction was determined by the BCS method (Pierce, Rockville, IL) using bovine serum albumin as standard. Protein samples (20 µg) from individual hearts were electrophoresed on 4–15% SDS–PAGE gels essentially as we have previously described [39], [40]. Briefly, separated proteins were transblotted onto nitrocellulose membranes, blocked for non-specific protein binding using 5% dry milk 50 mM Tris, pH7.2/150 mM NaCl/0.5% Tween 20 (TBST) and then probed for selected proteins with specific primary antibody followed by secondary peroxidase labeled antibody for detection. The specific primary antibodies used were rabbit anti- Hsp 47 (1∶5,000; MBL international), rabbit anti-Stathmin (1∶2,000 dilution; Sigma), rabbit anti- Desmin (1∶5,000 dilution; Sigma) goat anti-rabbit-HRP (1∶10,000 dilution; Jackson immunoResearch) secondary antibody (Sigma). Immunopositive bands were detected using ECL (Pierce). Multiple exposures of x-ray film were made with the ECL treated membrane to assure that the developed band densities were within the linear range of the film. Imaging was performed using an imaging camera system (Chemimager 4400; AlphaInotech) with no auto-gain [41]. Measurements to determine the relative densities were normalized to Desmin using NIH image J [42]. The relative density measurements obtained from 5 individual homozygous hearts were averaged and analyzed with Prizm 5 (GraphPad software, Inc).

## Results

### Septal Defects in the *Vcan* ^(tm1Zim)^ Mice

**Figure 2:**
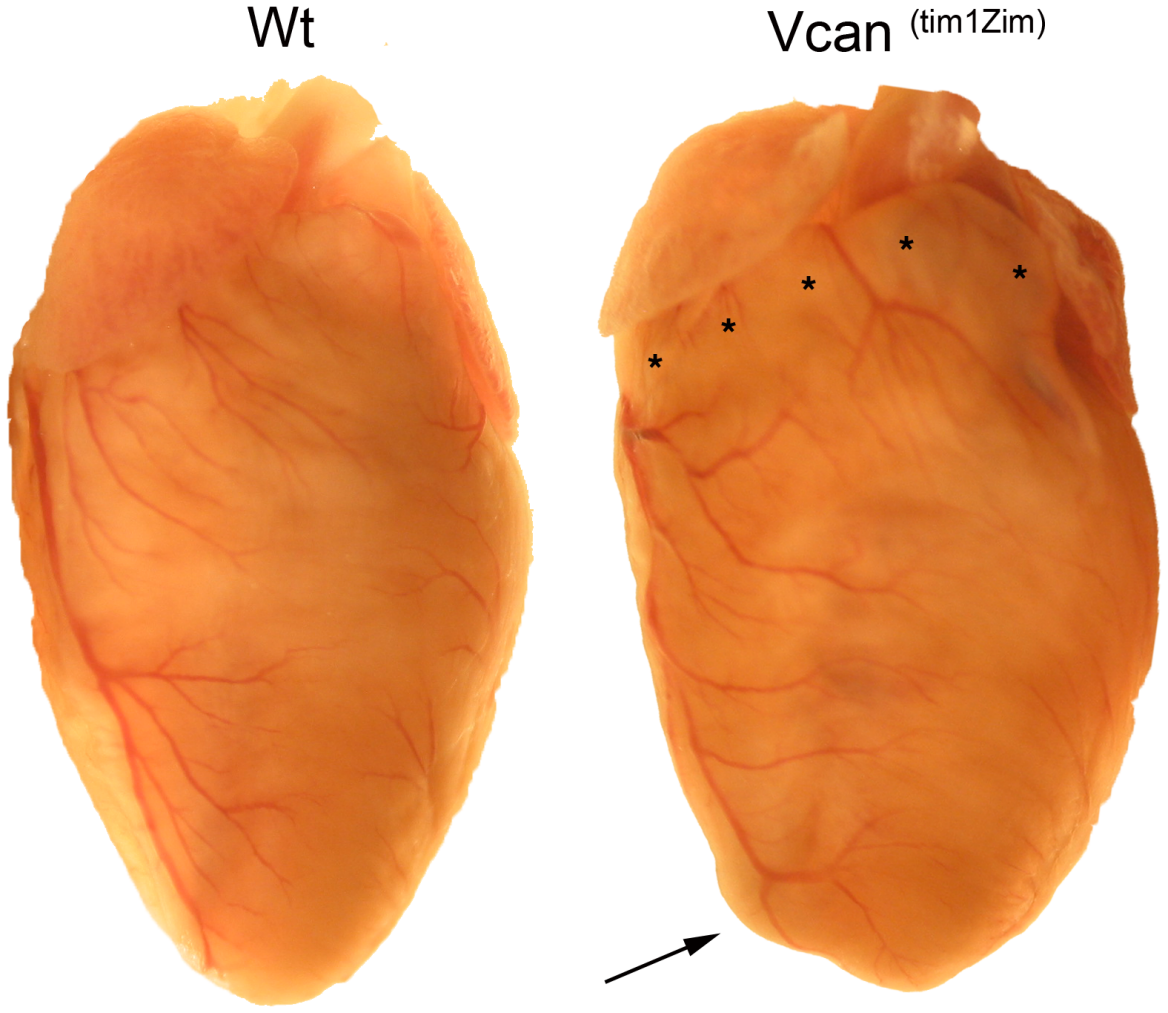
Morphological comparison of *Vcan* ^(tm1Zim)^ with wild-type adult hearts. The postnatal *Vcan* ^(tm1Zim)^ hearts showed a dilated right anterior ventricular wall that was most pronounced in the subpulmonary infundibulum adjacent to the right atrium (asterisks). The dilated right ventricle gave the apex of the *Vcan* ^(tm1Zim)^ hearts a more rounded external appearance than in wild-type hearts (arrow).

Gross and histological examination of the *Vcan* ^(tm1Zim)^ hearts showed an altered external morphology and the presence of heart defects. External examination of the postnatal *Vcan* ^(tm1Zim)^ hearts showed a dilated right ventricular wall that was most pronounced in the subpulmonary infundibulum adjacent to the right atrium (Fig. 2; asterisks). The dilated right ventricle also gave the apex of the *Vcan* ^(tm1Zim)^ hearts a more rounded external appearance than in wild-type hearts (Fig. 2; arrow).

**Figure 3:**
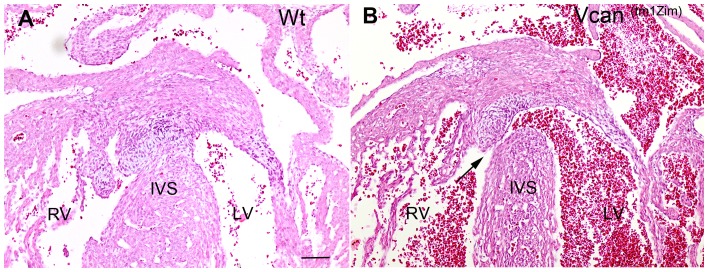
Interventricular septal defects found in the *Vcan* ^(tm1Zim)^ postnatal hearts. An example of IVS defect in the *Vcan* ^(tm1Zim)^ postnatal day 1 mouse heart (panel B; arrow) associated with a smaller mesenchymalized cushion then those in the wild-type (panel A) at the apex of the interventricular septum (IVS). RV-right ventricle; LV-left ventricle; IVS-interventricular septum. Magnification bar in panel A = 200 µm and is the same for panel B.

**Figure 4:**
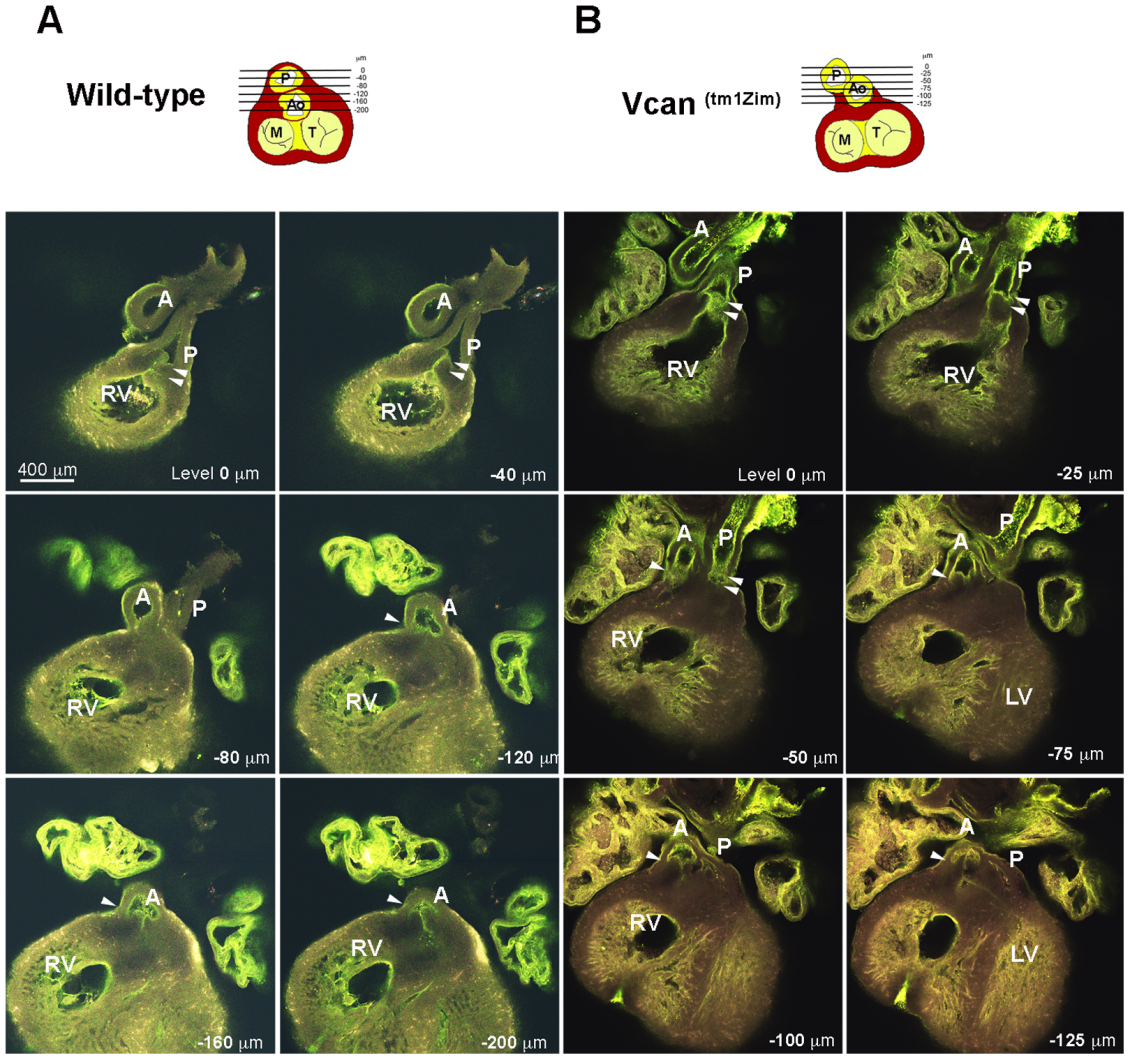
Whole-heart confocal optical imaging of defects in *Vcan* ^(tm1Zim)^ E13.5 dpc mouse hearts. In the wild-type (Panel A), consecutively deeper optical section images showed the pulmonary outlet is ventral to the aortic outlet at the base of the heart. In the *Vcan* ^(tm1Zim)^ (Panel B), the aorta and pulmonary outlets were within the same plane of optical section due to altered/defective outflow tract rotation and integration (−50 um). Hearts were treated to increase optical transparency and then perfused with FITC conjugated poly-L-lysine to visualize the endothelial lining of the vessels (yellow/green). Each optical section with each panel is a frontal image taken at sequentially deeper focal planes. Panels for the wild-type heart (panel A) span a total depth of 200 µm. The *Vcan* ^(tm1Zim)^ heart (panel B) required imaging through a much shorter distance (125 µm) to image the same structures. RV-right ventricle; A-aorta; P-pulmonary; M-mitral; T-tricuspid.

Histological examination showed that many of the homozygous *Vcan* ^(tm1Zim)^ hearts achieved normal internal septation. However, AV cushions in all the *Vcan* ^(tm1Zim)^ hearts were smaller than those found in the wild-type hearts (see below) and ventricular septal defects were observed (Fig. 3; 37%; 14 of 38 postnatal day 1 hearts). Also, an abnormal relationship was observed between the aortic and pulmonary roots in the mutant hearts. Combined, these structural defects suggested developmental alterations in the mutants at or around embryonic stage 13.5 pc when there is a closure of membranous portion of the interventricular septum and the cardiac outlet is integrated into the central cushions [43]. To explore this hypothesis, we used confocal optical sectioning of E13.5 dpc hearts that were rendered semi-transparent to compare the relative orientation of the aortic and pulmonary roots at sequentially deeper Z-planes. Using this technique in the wild-type heart, we found the pulmonary outlet was imaged in a more ventral plane of optical section (Fig. 4A, Z-plane 0 to −40 µm) relative to the aortic outlet that appeared in a deeper Z-plane level (Fig. 4A, Z-plane −120 to −200 µm) as expected for a normal mouse heart. However, imaging of the mutant heart showed that both the pulmonary and aortic roots are abnormally found within the same Z-plane of optical section (Fig. 4B, Z-plane −25 through −50 µm). The homozygous phenotypes were consistent with a common underlying problem occurring in the final developmental stages of the outflow tract’s integration into the base of the heart that is occurring at E13.5 pc in the mouse.

### Altered Atrioventricular and Outflow Tract Structures in the *Vcan* ^(tm1Zim)^ Hearts

**Figure 5:**
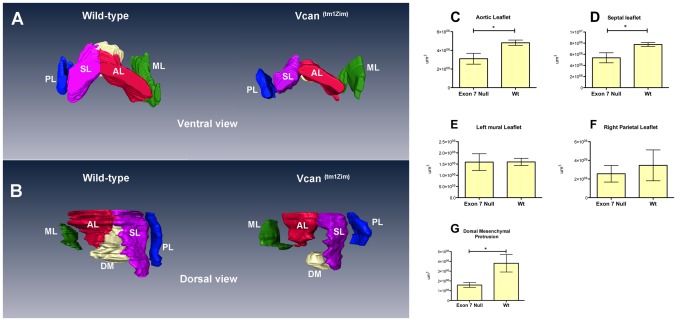
Three-dimensional reconstructions and quantitative measurements of wild-type and *Vcan* exon 7 AV associated cushions in E13.5 pc hearts. The differences found in the mesenchymalized AV cushions, visually apparent in the 3-dimensional comparisons (Panel A, ventral view; B, dorsal view), were also quantified using the AMIRA imaging software to measure the cushion volumes. A significant reduction in volume was measured in the central AV cushion comprising the aortic leaflets (AL in panels A, B; reduced 35%; *p<0.034 n = 3 for each genotype, panel C) and septal (SL in panels A,B; reduced 30% p<0.046 n = 3 for each genotype in panel D). A significant (*p value 0.05 panel G) decrease in volume (0.58x) of the dorsal mesenchymal protrusion (DM) was measured. The other cushions showed no significant difference and the overall size of the E13.5 *Vcan* ^(tm1Zim)^ and wild-type hearts (measured by tissue weight) was not significantly different (74 mg and 75.6 mg respectively; n = 3 for each genotype). AL-aortic leaflet (red); PL-parietal leaflet (blue); SL-septal leaflet (pink); ML-mural leaflet (green); DM-dorsal mesenchyme (white).

**Figure 6:**
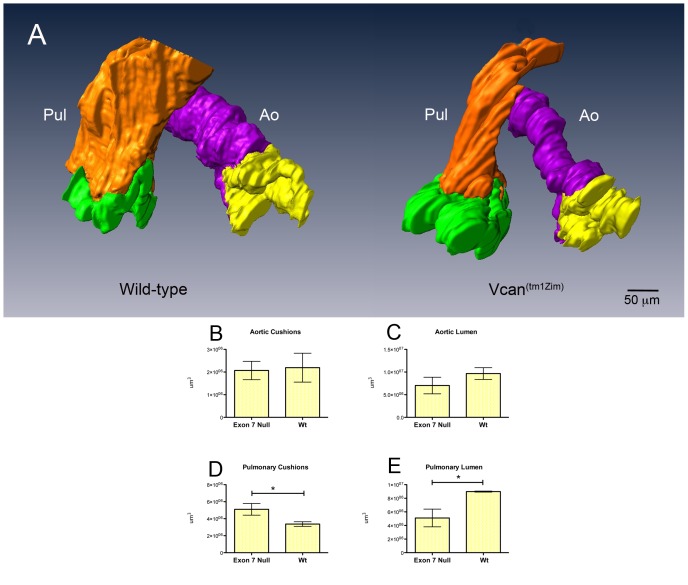
Three-dimensional reconstruction and quantitative comparisons of wild-type and *Vcan* exon 7 cardiac OT associated cushions in E13.5 pc hearts. Significant differences were found in the volume of the pulmonary lumen and size of the pulmonary cushions in the *Vcan* ^(tm1Zim)^ compared to wild-type littermates. The differences, visually apparent in the 3-dimensional comparisons (panel A; wild-type and *Vcan* ^(tm1Zim)^), were quantified using the AMIRA imaging software to measure the volumes of the cushion primordia of the pulmonary and aortic valves and of the pulmonary (brown) and aortic (purple) lumens. A significant increase (51%; *P value 0.047 panel D) was found in the size of the pulmonary cushions in the *Vcan* ^(tm1Zim)^ hearts compared to wild-type littermates (panels D). The pulmonary lumens of the *Vcan* ^(tm1Zim)^ cardiac outlets were found to be significantly (panel E; *P value 0.028 ) smaller (57%) then that of wild-type littermate controls. The aortic lumen volumes measured smaller but did not show a significant change (panel C). Brown-pulmonary lumen (PL); purple-aortic lumen (Ao); green-pulmonary cushions; yellow-aortic cushions.

**Figure 7:**
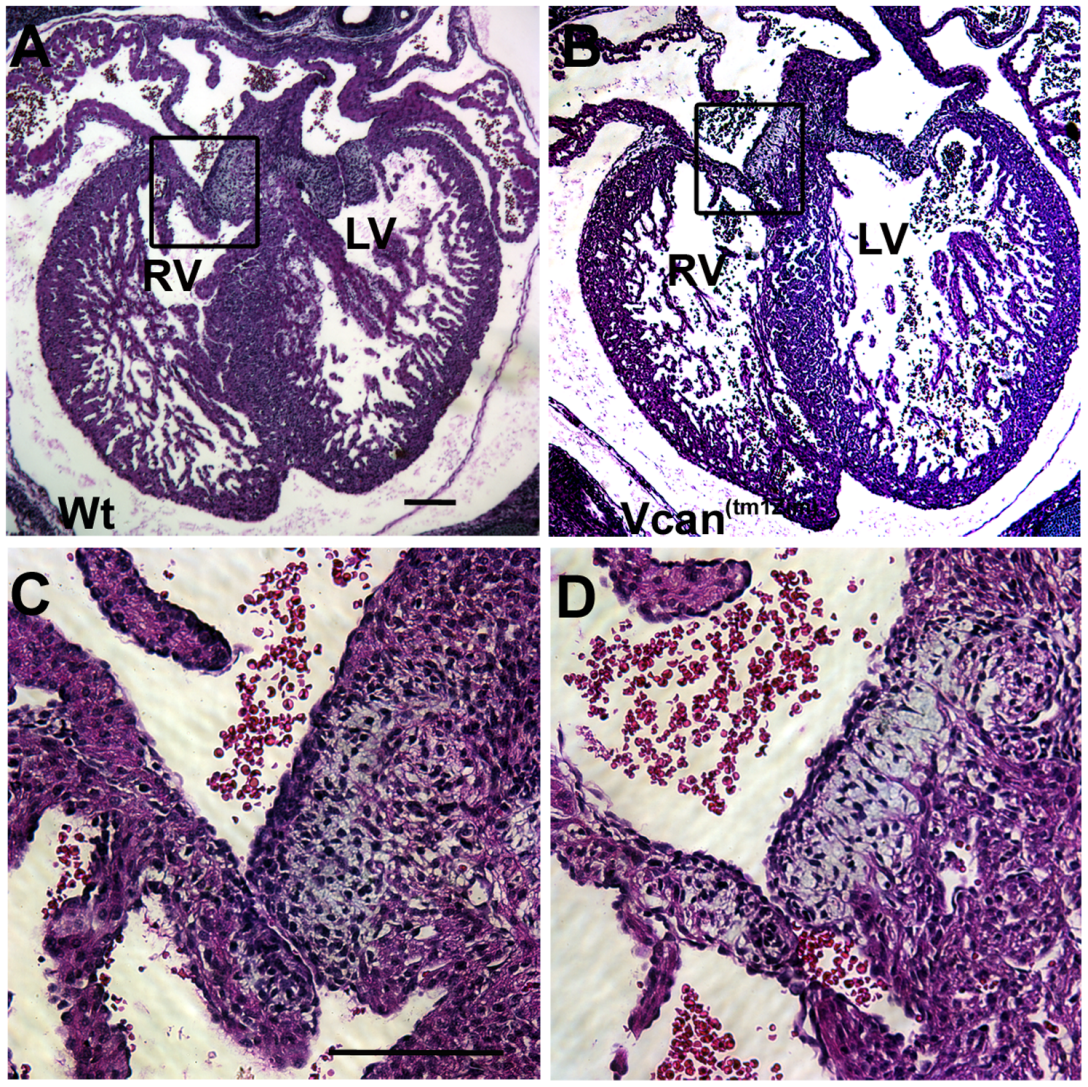
Histological comparison of cushions in the *Vcan* ^(tm1Zim)^. Hematoxylin/eosin stained sections of postnatal day 1 (B, D) and *Vcan* ^(tm1Zim)^ (A, C) hearts were compared. Boxed region in A and B of the cushion is shown higher magnification in C and D. Note the smaller cushions in the *Vcan* ^(tm1Zim)^ heart. Panel A and B are the same magnification as are C and D; magnification bars = 200 µm.

**Figure 8:**
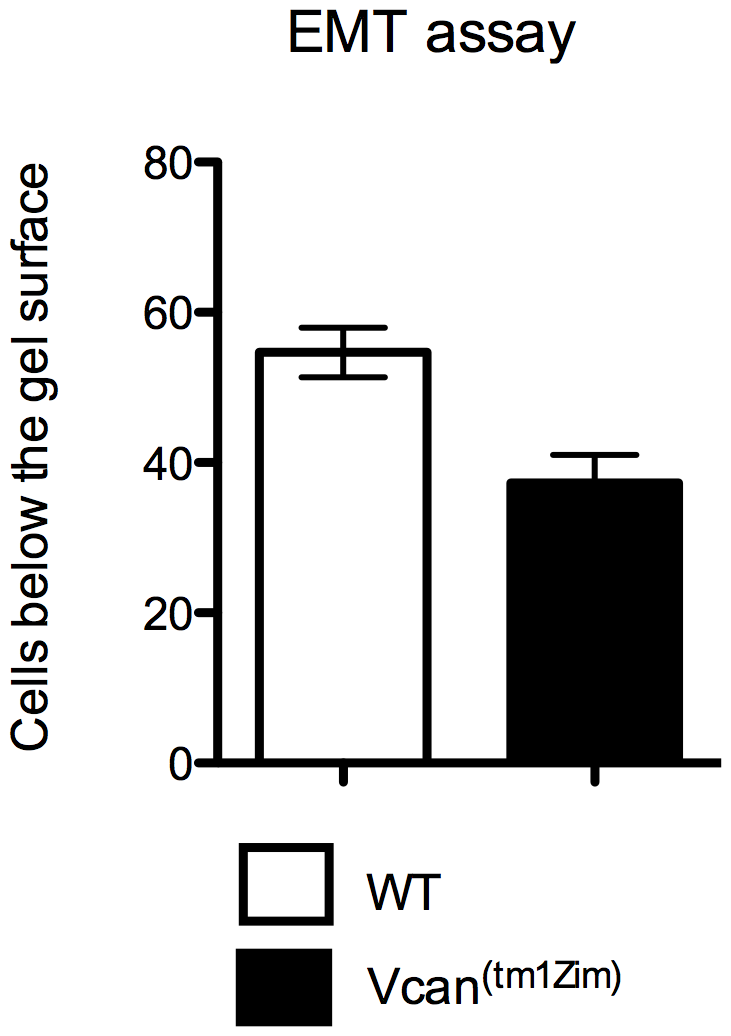
Epithelial-mesenchymal 3D gel assay. Atrioventricular cushion explants from E10.5 pc hearts cultured on 3D hydrated collagen gels in the EMT assay produced significantly (p<0.05) less numbers of mesenchymal cells found below the surface of the gel from explants of *Vcan* ^(tm1Zim)^ mice (average 37/explant; n = 3) vs. wild-type littermate controls (average 55/explant; n = 3).

Histological three-dimensional (3D) reconstructions of the *Vcan* ^(tm1Zim)^ hearts at E13.5 pc showed significant differences in the size of the central AV endocardial cushions of the heart (Fig. 5A,B). Both the mesenchymalized aortic and septal leaflet cushions of the AV canal were significantly reduced in total volume in the *Vcan* ^(tm1Zim)^ hearts as determined by AMIRA 3D reconstruction measurements (Fig. 5C,D). Similar measurements of the parietal leaflet of the right AV and the mural leaflet of the left AV showed no significant difference in total volume between *Vcan* ^(tm1Zim)^ and wild type littermates (Fig. 5E, F). The dorsal mesenchymal protrusion was smaller in volume and did not integrate fully into the central AV cushion complex (Fig. 5B,G). In the cardiac outlet, measurements of the forming pulmonary leaflet cushions showed a significant (51%) increase in total volume in the *Vcan* ^(tm1Zim)^ (Fig. 6A, D). Whereas, the total volume occupied by the cushions of the aortic leaflets were of similar size (Fig. 6A,B). In addition, the lumen of the pulmonary artery is significantly smaller in the *Vcan* ^(tm1Zim)^ compared to the wild-type animals (Fig. 6A, E) The lumen of the ascending aortic artery showed no significant difference between *Vcan* ^(tm1Zim)^ and wild-type animals (Fig. 6A,C). In newborn (postnatal day1) hearts, the *Vcan* ^(tm1Zim)^ leaflets also appeared smaller than those found in the wild-type littermate controls (Fig. 7). The density of mesenchymal cell nuclei in the AV cushions was reduced in the *Vcan* ^(tm1Zim)^ hearts (62% of wild-type; average of 8 nuclei/160 um^2^ vs. 13 nuclei/160 um^2^ respectively; SD = 1.8; n = 4; p = 0.01). Cushion explants from the AV regions of *Vcan* ^(tm1Zim)^ hearts cultured on 3D hydrated collagen gels (EMT assay) [29], [30] produced fewer mesenchymal cells below the surface of the gel compared to wild-type littermate controls (Fig. 8).

### Localization of Extracellular Matrix (ECM) Molecules in the Adult Valvular Cushions and Great Arteries

**Figure 9:**
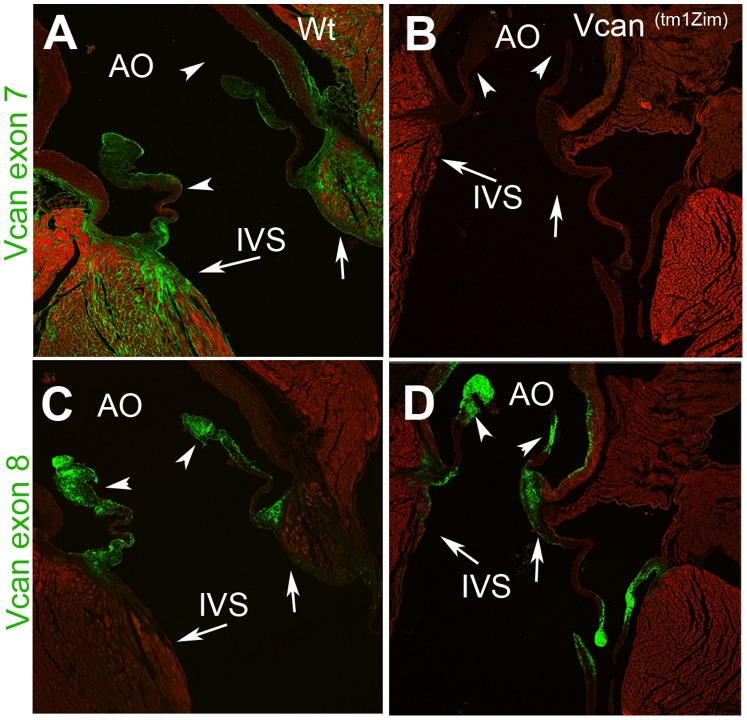
Comparisons of versican domains in the *Vcan* ^(tm1Zim)^ and wild-type adult hearts. Immunohistological localization of the GAGα antibody (epitope found in exon 7) showed no immunofluorescence in the *Vcan* ^(tm1Zim)^ heart (B). GAGα antibody in wild-type hearts (A) localized to the aortic walls and valves (arrowheads), interstitial tissue of the interventricular septum (IVS) and of the aortic mitral continuity (arrow). The GAGβ antibody (epitope found in exon 8) was localized in the *Vcan* ^(tm1Zim)^ (D) to the aorta walls, valvular leaflets (arrowheads), IVS and aortic mitral continuity, but GAGβ positive cells were not found interspersed between the sarcomeric myosin positive cells (arrow) of the aortic mitral continuity as seen in the wild-type heart (E). Within the wild-type interstitial tissue of the interventricular septum myocardium and aortic mitral continuity GAGβ localization was relatively absent compared to the GAGα localization in these regions (compare IVS- arrows in A and C; identical confocal settings). The magnification bar in panel A = 200 µm applies to all panels. Ao-aorta; IVS-interventricular septum.

Versican is prominently expressed in the mature cardiac valves, and the great arteries [44]–[46]. To morphologically assess any detectable changes in versican and other specific ECM markers that result from the loss of the V2/V0 splice forms we evaluated the structures in adult hearts (5 ½ weeks postnatal) by confocal indirect immunofluorescence. Antibodies recognizing either exon 7 (GAG-α) or exon 8 (GAG-β) domains of versican were used to differentiate between the expression of the full length and alternative splice forms V1 and V2. Both antibodies recognize the V0 splice form, however, it is absent in the *Vcan* ^(tm1Zim)^ mouse. Results confirmed that, as expected, the GAG-α/exon 7 antibody did not react with the *Vcan* ^(tm1Zim)^ hearts since the polypeptide region containing the epitope recognized by this antibody is missing in the *Vcan* ^(tm1Zim)^ mouse (Fig. 9B). Localization in wild-type hearts of the GAG-α/exon 7 epitope was most intense along the ventricular side of the aortic leaflets plus the luminal and external regions of the aortic wall. Strong localization was also found in the interstitium of the heart myocardium within the interventricular septum (IVS) near the hinge region of the valvular leafets, the aortic mitral continuity and atrial myocardium (Fig. 9A).

Immunolocalization of the GAG-β/exon 8 epitope, found in both V0 and V1 versican, was localized throughout all valvular leaflets in both the wild-type and the *Vcan* ^(tm1Zim)^ hearts (Fig. 9C&D). However, little or no staining was seen within the interstitium of the myocardium (e.g., interventricular septum) for this eiptope in either wild-type or *Vcan* ^(tm1Zim)^ hearts.

**Figure 10:**
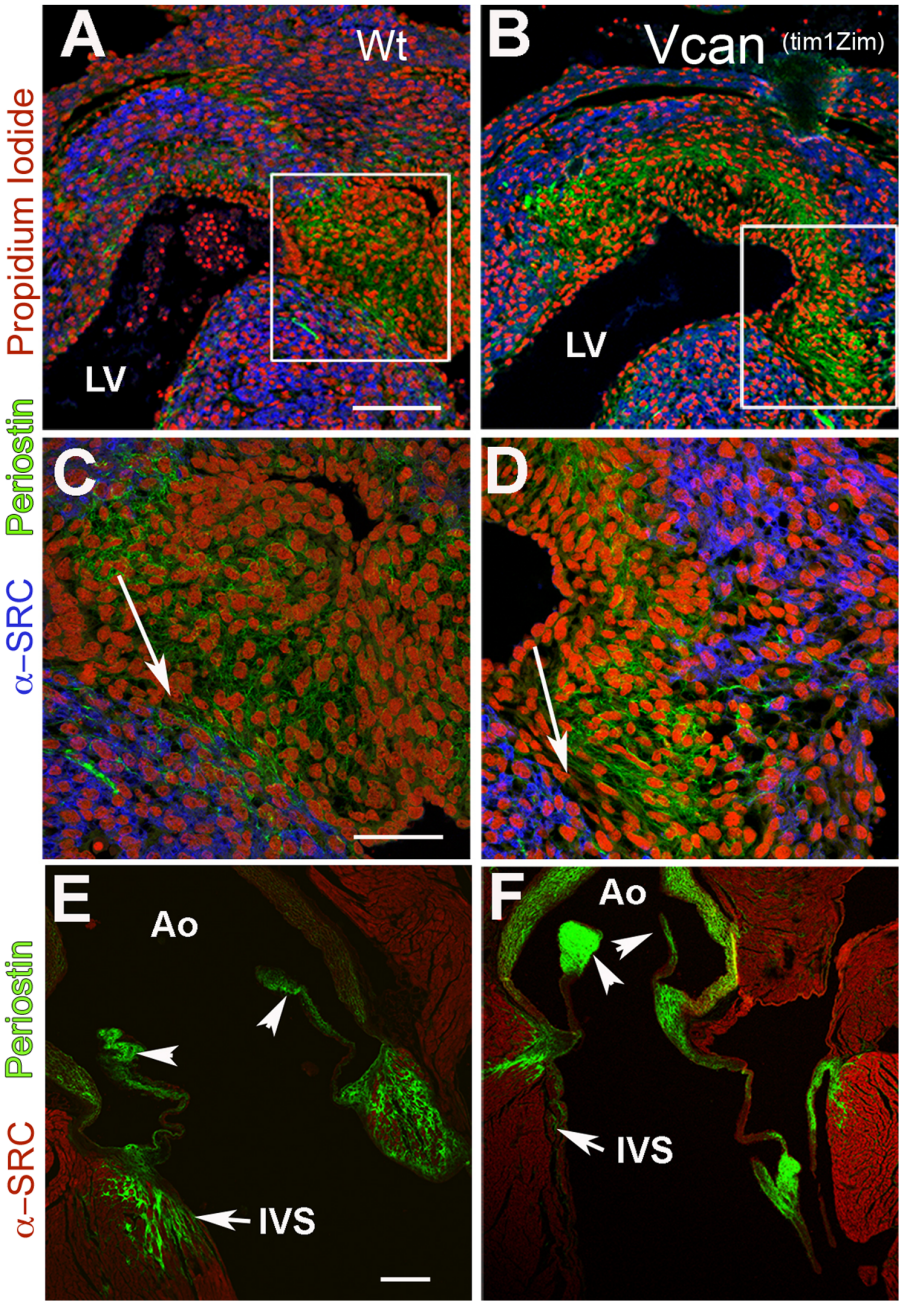
Comparison of periostin in the cushions and valves of *Vcan* ^(tm1Zim)^ embryonic and adult hearts. Comparisons of periostin expression in wild-type E13.5 pc hearts (panels A, C) and *Vcan* ^(tm1Zim)^ E13.5 pc hearts (panels B,D) by confocal immunofluorescent localization. Periostin (green) expression in the *Vcan* ^(tm1Zim)^ hearts appeared more compact and less widely distributed throughout the cushions than in the wild-type. Boxes in panels A,B denote magnified areas shown in C,D. Periostin staining showed a sharper boundary between the apex of the interventricular septum (blue; α-sarcomeric myosin) and the adjacent cushion mesenchyme in the *Vcan* ^(tm1Zim)^ (D; arrow) as compared to that observed in wild-types (C; arrows). In adult hearts, periostin expression in the *Vcan* ^(tm1Zim)^ (F) appeared more intense in the aorta (Ao) walls and valve leaflets, however very little or no localization of periostin was observed in the interstitial tissue of the muscular interventricular septum (IVS) as compared to wild-type (E). In the *Vcan* ^(tm1Zim)^ heart, periostin appeared to localize only in zones adjacent to the myocardium (F;IVS), but did not show an interspersed pattern of expression between individual myocytes as seen in the wild-type IVS and aortic mitral continuity(E;IVS). Panels A & B same magnification bar = 150 µm; panels C & D same magnification bar = 50 µm; panels E & F same magnification bar = 200 µm.

We hypothesized that the exon 7 containing variants of Vcan may participate in the organization of other ECM molecules. To further assess alterations in the *Vcan* ^(tm1Zim)^ extracellular matrix the protein periostin was evaluated in embryonic and adult hearts because it is expressed in a close pattern with versican in developing cushions and adult structures [19], [45]. We chose E13.5 dpc developmental stage, since this is an embryonic stage that exon 7 epitope is clearly detectable at the protein level. Figure 9, shows the confocal immunofluorescence image comparisons of periostin, in embryonic (E13.5) and adult *Vcan* ^(tm1Zim)^ and wild-type hearts. Staining for periostin appeared more abundant and fibrillar in the *Vcan* ^(tm1Zim)^ (Fig. 10 B,D) than observed in wild-type (Fig. 10 A,C) endocardial cushions of E13.5 pc embryonic hearts. In the wild-type adult heart periostin staining localized to the aortic wall, valve leaflets, and the myocardial interstitial tissue of aortic mitral continuity (Fig. 10E). Staining was also apparent in interstitial tissues of the myocardium at the base of the aortic valve leaflet in the interventricular septum of the wild-type hearts (Fig. 10E). In the *Vcan* ^(tm1Zim)^ hearts, periostin was detected in a similar pattern, but with more intensity in the walls of the aorta (Fig. 10F). Also, the interstitial localizations of periostin in the interventricular septum showed a more restricted pattern of localization between the myocardial cells in the *Vcan* ^(tm1Zim)^ hearts (compare IVS of wild-type and mutant in Fig. 10E,F). Overall, the loss of the Vcan variant appears to alter periostin’s deposition pattern in the matrix.

### *Vcan^(^* ^tm1Zim)^ Hearts have Deficiencies in Cushion Tissue Muscularization

**Figure 11:**
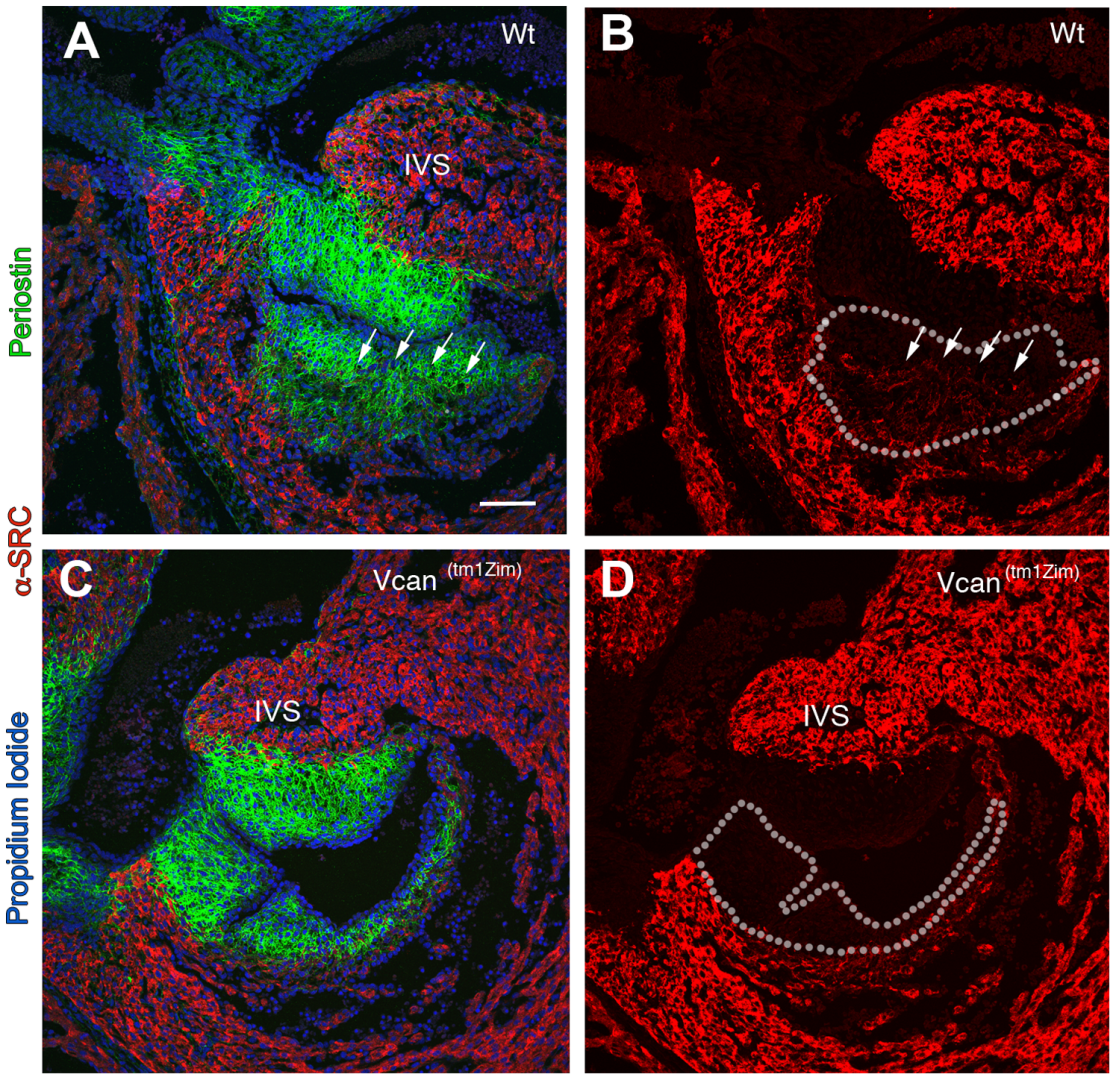
Restricted or delayed process of myocardialization observed in the *Vcan* ^(tm1Zim)^ hearts. E13.5 pc wild-type hearts (A, B) compared to *Vcan* ^(tm1Zim)^ (C, D). The image in each panel is one taken from sections through the right parietal cushions at their thickest margin. Panels A and C utilize both periostin staining (green) and α-sarcomeric actin (red) to mark the cushion and muscle boundaries (propidium iodide marks the cell nuclei in blue). Panels B and D are the same image as A and C with periostin and propidium iodide staining removed to show the extent of muscle cells (α-sarcomeric actin positive) present within the cushion more clearly (A & B; arrows mark the muscle boundary; dotted lines mark the boundaries of the cushion). The WT hearts show that muscle cells are present in the cushion. The *Vcan* ^(tm1Zim)^ hearts show little or no presence of muscle into the adjacent matrix of the phenotypically smaller cushions of the *Vcan* ^(tm1Zim)^ hearts (B, C). IVS-interventricular septum. Magnification bar in panel A = 100 µm and applies to all panels.

**Figure 12:**
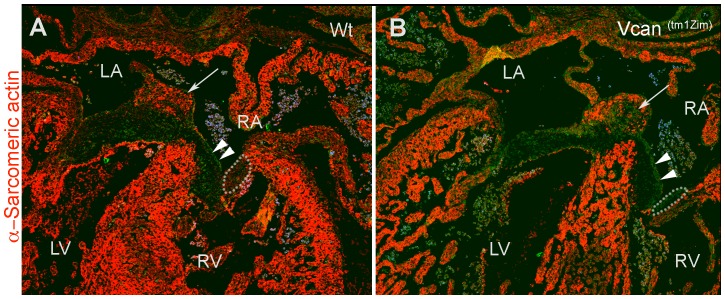
Four-chambered view of the wild-type (A) and *Vcan* ^(tm1Zim)^ E13.5 hearts (B) show a diminished level of α-sarcomeric actin staining (arrow in A & B) in the mesenchyme of the central AV cushion consisting of the dorsal mesenchymal protrusion and spina vestibule as compared with the wild-type hearts (B). A complete lack of α-sarcomeric actin staining was observed in the cushion of the right AV parietal leaflet cushion in the *Vcan* ^(tm1Zim)^ (B; within the area bounded by the dotted line), but α-sarcomeric actin staining was clearly present in the same leaflet cushion of the wild-type (A; within the dotted line). Double arrowheads in A and B denote the septal AV leaflet cushion. RA-right atrium; LA-left atrium; RV-right ventricle, LV-left ventricle. Magnification bar 100 µm.

Immunohistological analysis of the *Vcan* ^(tm1Zim)^ hearts (E13.5 pc) showed a lack of cells positive for α-sarcomeric actin within the mesenchyme of the right parietal leaflet and reduced amounts in the central mesenchymalized cushions (Fig. 11). Regions of the endocardial cushions *Vcan* ^(tm1Zim)^ become muscularized during the late stages of embryonic development through a process of differentiation or myocardial cell invasion [47], [48]. In the *Vcan* ^(tm1Zim)^ E13.5 hearts, cells positive for of α-sarcomeric actin staining are completely absent within the cushion mesenchyme of the right parietal leaflet (Fig. 11 A,B,C,D and Fig. 12 ). Also the *Vcan* ^(tm1Zim)^ hearts have a diminished level of α-sarcomeric actin staining in the central regions of mesenchyme formed by the spina vestibule and dorsal mesenchymal protrusion (Fig. 12 B; single arrow).

### Proteomic iTRAQ Analysis of the *Vcan* ^(tm1Zim)^ Hearts

To identify some of the resulting differences in cellular protein abundance that results from the loss of the extracellular *Vcan* splice forms, a high-throughput quantitative proteomic analysis (iTRAQ; Applied Biosystems) was used. Protein extractions were made of separately pooled E13.5 pc *Vcan* ^(tm1Zim)^ (n = 5) hearts and wild-type (n = 5) hearts. Replicate aliquots of the WT and *Vcan* ^(tm1Zim)^ heart homogenates were labeled with unique isobaric tags for separate analysis. The separate protein fractions were then analyzed by iTRAQ to profile the identities and relative changes in abundance. The replicate aliquots from the same groups allowed for controlled comparisons within each group. Therefore, as an internal control the ratio of protein abundance for each identified protein within the same (*Vcan* ^(tm1Zim)^ or wt) replicate aliquot should yield a value of 1.0. Only those that deviated from a ratio of 1.0 were considered to represent a unique protein fingerprint for *Vcan* ^(tm1Zim)^ hearts compared to wild-type. Identities, relative abundance and statistical analysis of multiple peptides mapping to individual proteins were performed on the data using methods and software previously described [35].

**Figure 13:**
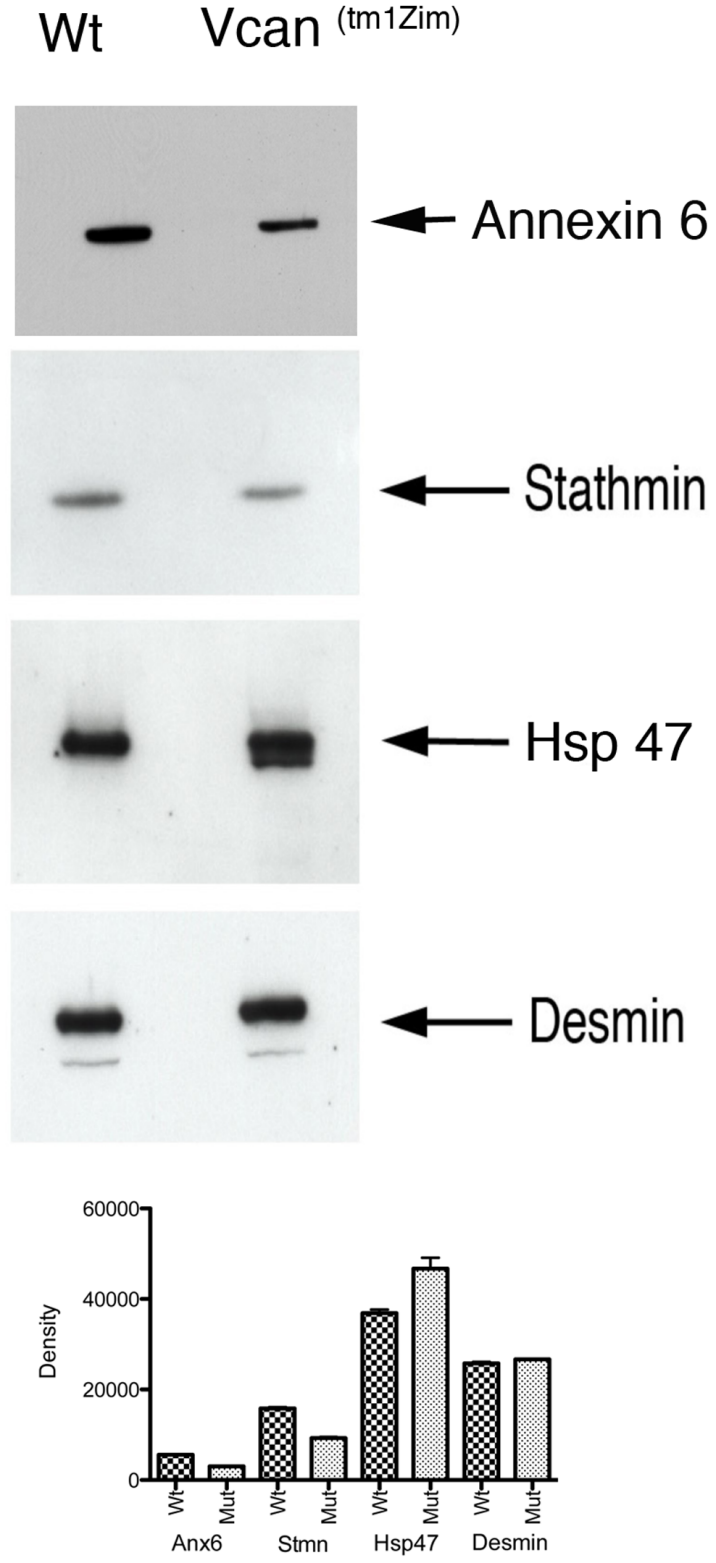
Western blot comparison of four proteins with altered abundance by itraq in *Vcan* ^(tm1Zim)^ mutant and wild-type hearts. Proteins that showed different levels of altered abundance in the *Vcan* ^(tm1Zim)^ hearts are shown in the blot. Annexin A6 and Stathmin are proteins expressed in the heart that showed a relative decrease (0.85x and 0.73x respectively) in abundance in the *Vcan* ^(tm1Zim)^ mutant by iTRAQ and serphin1 (Hsp 47) that by iTRAQ showed increased abundance (1.55x). Additionally, Desmin that did not change with any significance between mutant (Mut) and wild-type (Wt) by iTRAQ, showed no significant change by western blot. Results of average relative density measurements of separately analyzed hearts are shown in the graph for each protein (for Stmn, Hsp47, Desmin n = 3; p<0.01; for Annexin A6 n = 2; p<0.0045).

A total of 5095 redundant iTRAQ pairs were identified for the *Vcan* ^(tm1Zim)^ and WT fractions. A total of 2882 unique peptides were identified, corresponding to 940 proteins. Well over 50% of the proteins identified were represented by two or more unique peptides. Only those proteins with at least two unique peptides are reported. Forty-seven proteins showed significant increases in the mean expression ratio of abundance by more than 1.20-fold in the *Vcan* ^(tm1Zim)^ mouse heart and 74 were decreased by more than 0.85X (File S1). The relative change in protein abundance measured by iTRAQ was verified for selected proteins of interest by western blots of E13.5 pc staged heart extracts. As predicted, the relative abundance of Vcan in the *Vcan* ^(tm1Zim)^ hearts was reduced (0.45x) in abundance compared to the wild-type. Other proteins of interest were surveyed in western blots to verify the relative change in abundance detected by iTRAQ. For example, Annexin A6 (4 unique peptides) and Stathmin 1 (9 uniques peptides) together showed a relative decrease (0.85 and 0.73 respectively) in the *Vcan* ^(tm1Zim)^ heart. Also, all 3 peptides mapped to Serpinh1 (Hsp 47) showed an increased abundance (1.55) in the *Vcan* ^(tm1Zim)^. The relative decreases or increase was confirmed by western blots of protein in more than one animal (Stathmin, Hsp 47 and Desmin, n = 3; Annexin A6, n = 2; Fig. 13). The relative changes in density were significant and normalized in the immunoblot to Desmin that measured with a mean expression ratio average of 0.92 for all 22 peptides identified. We sorted the proteins found to change in abundance in the *Vcan* ^(tm1Zim)^ hearts into standard gene ontology (GO) categories (File S2). The gene ontology categories for altered protein levels showed that the loss of *Vcan* exon7 alters cellular processes at multiple levels including those that would likely underlie the observed heart and vasculature phenotype. These include altered abundance of proteins needed in extracellular matrix assembly, signal transduction, muscle differentiation/function, apoptosis and proliferation.

## Discussion

Since the initial identification and characterization of versican [3], the functional importance of this chondroitin sulfate proteoglycan in multiple adult biological systems has been increasingly demonstrated [49]–[51]. Similarly, the expression pattern of versican in embryogenesis and analysis of the insertional mutant mouse *hdf* have shown that versican is indispensable during the early formation of the heart [5], [52] and at later stages is needed for closure of the interventricular foramen [22], [32]. The expression of alternative splice forms, within specific cardiac tissue domains in the later stages of heart development has suggested that versican splice forms may play important specific roles in the later embryonic and fetal stages of heart formation. In this study, we show a requirement for exon 7 containing versican splice forms V0/V2 during normal heart development at later stages that impact the mature heart structure. Detailed analyses of the *Vcan* ^(tm1Zim)^ mice revealed septal defects, changes in the size of some mesenchymalized cushions, dilated right ventricle with dilation of the sub-pulmonary infundibulum and altered integration of the aortic and pulmonary trunks into the AV cushion complex. These combined altered phenotypes result in a failure of the outflow tract to integrate and align correctly with the ventricles during development.

We observed the presence of VSDs in the Vcan^(tm1Zim)^ hearts. Closure of the interventricular foramen occurs at approximately E13–14 p.c. in the wild-type mice on the C57B6 background. In the E13.5 p.c. hearts, analyzed by AMIRA 3d reconstruction, forming cushions of the dorsal mesenchymal protrusion, septal and aortic leaflets were significantly smaller and did not merge as in wild-type. This reduction in cushion tissue could contribute to the higher incidence of VSD at E13.5, however many of these defects eventually close as was observed shortly after weaning of the newborn mice (5.5 weeks PN; 37% VSD). The presence of ventricular septal defects have been described in other mouse models of *Vcan* partial depletion that include the *Crtl-1* null, *hdf* heterozygous and the *Vcan* subdomain A null mouse (*Vcan^Δ3/Δ3^*) [22]. Comparison of *Vcan^(tm1Zim^)* exon mouse heart defects with the Crtl1-null and the *hdf* heterozygote models show this overlap in structural defects (e.g. VSD) [32], but also an additional spectrum of cardiac outlet defects in the *Vcan^(tm1Zim)^* reported here. We also know that *hdf* heterozygous [32] and *Vcan^(tm1Zim)^* heart have reduced levels (approximately 50%) of versican. Similarly, the expression of the truncated *Vcan* gene in *Vcan^Δ3/Δ3^* is approximately 55% of the wild type, The *hdf* heterozygote is able to express all splice forms of Vcan from its unaffected allele, the *Vcan^ Δ3/Δ3^* expresses an altered form containing the G3 domain and the *Vcan^(tm1Zim)^* can only expresses 2 of the 4 alternative splice form variants. Taken together the observations suggest that the VSDs observed in all 3 models are the result of an overall reduction in versican expression and not specifically due to the splice variants expressed.

**Figure 14:**
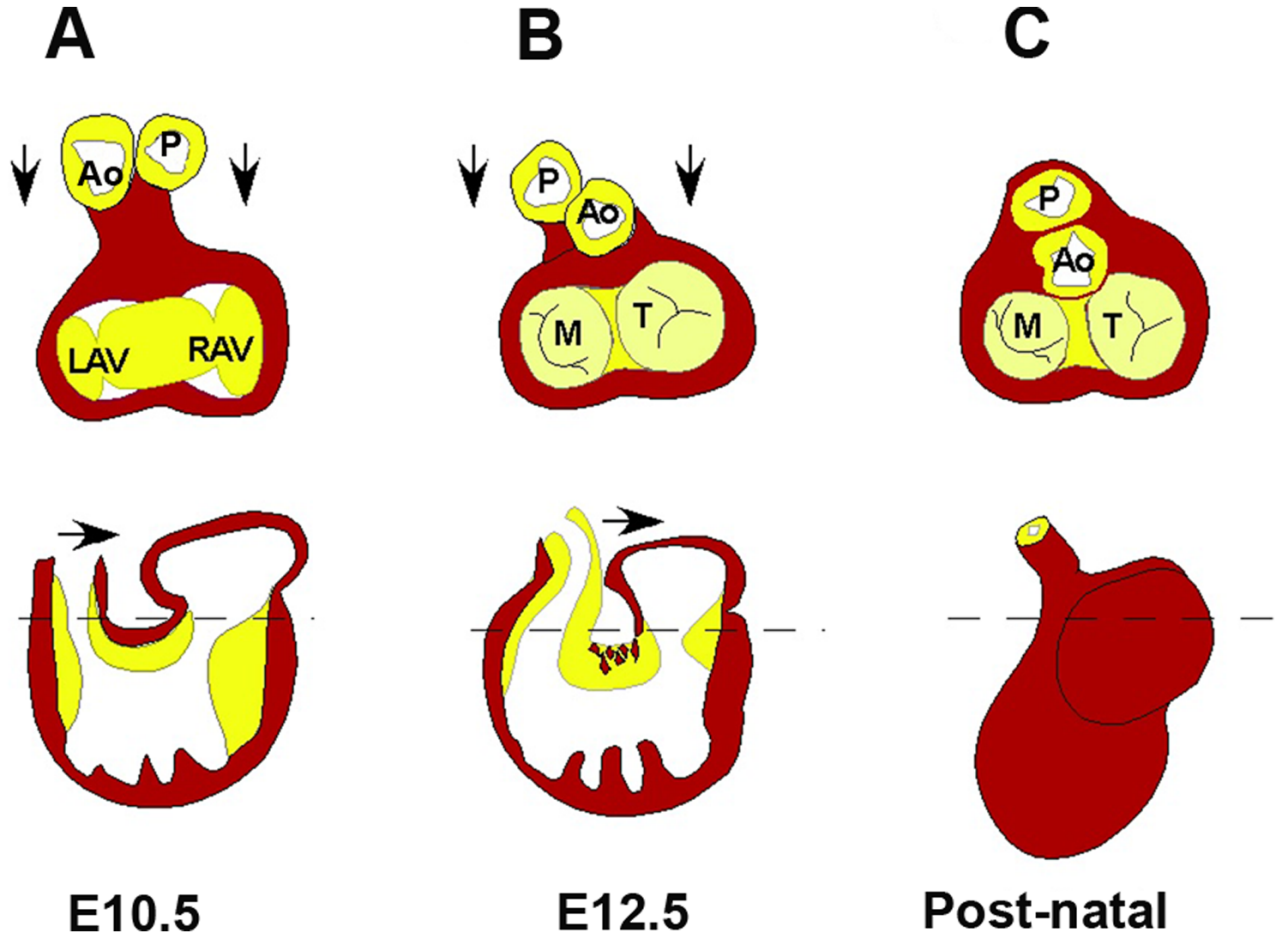
Normal rotation and integration of the outflow tract (cardiac outlet) into the fused atrioventricular (AV) cushion in the final stages of looping and septal alignment. Upper panels are cross section dorsal views showing left and right AV canals, aorta and pulmonary trunks (Ao & P). Lower panels are depictions of the sagittal view. The dotted line indicates approximate section shown in upper panels. The myocardium (*inner curvature) between the outlet and AV cushion is removed and myocardial cells invade the proximal outlet cushion as in E12.5. P-pulmonary artery, Ao-aorta, LAV & RAV, left & right atrioventricular canals, M-mitral valve, T-tricuspid valve.

The process by which the outflow tract becomes aligned with the ventricles involves the integration of the proximal outflow tract cushion into the central mesenchymalized cushion of the E13.5 p.c. heart. This positions the aortic and pulmonary arterial trunks in proper alignment between the left and right ventricular canals (Fig. 14). The mechanism through which this integration occurs is not well understood, however, part of the process involves the loss of inner curvature muscle and the appearance of cardiac muscle within the adjacent cushion mesenchyme. This process has been termed myocardialization [48], [53], [54]. The inner curvature musculature is removed by myocardialization during the final stages of looping that brings the proximal outlet segment septum (conus septum) into proper alignment with the ventricles of the 4-chambered heart. Changes in the size of mesenchymalized cushions and related changes in the myocardialization process has been observed in other models that have incomplete cardiac outlet integration e.g., the Vang12 null, [54], [55]Tgfß- 2 null [48], Trisomy 16 mouse [56], [57] and connexin 43 null [58].

Both the right mural and the central AV mesenchymalized cushions in *Vcan* ^(tm1Zim)^ heart show evidence of less myocardialization. The *Vcan* ^(tm1Zim)^ hearts at E13.5 pc showed reduced numbers of cells positive for α-sarcomeric actin within both these cushions (Fig. 11 & 12). This suggests a reduced ability of these cushions to undergo the normal process of myocardialization in the absence of *Vcan* splice forms V0/V2. This could be due to 1) a non-permissive matrix environment in the cushion that does not promote or prevents myocardial differentiation and/or invasion of needed precursor cells; 2) inability of the cushion matrix to sustain the myocardial phenotype within the cushion. The loss of *Vcan* exon 7 containing alternative splice forms V0/V2 may directly result in a “non-permissive” matrix, or indirectly alter other ECM molecules to interfere with invasion and/or myocardial phenotypic stability. We have shown that there are overall fewer cushion mesenchymal cells in the *Vcan* ^(tm1Zim)^ heart. It should be noted that the time points chosen for this study might not be entirely sufficient to fully understand the mechanisms of the valvular defects. Others have shown that a sufficient threshold level of cushion mesenchyme is essential for myocardialization to occur using in vitro assays that model the process [59]. The precise role of the cushion mesenchyme remains unclear, however, indications are that these cells produce an inductive signal (e.g., growth factor) that triggers the myocardial invasion [60]. However, a lack of myocardialization has been reported in both hypo-mesenchymal [56] and hyper-mesenchymalized heart cushions [48]. This suggests that the absolute number of total mesenchymal cells may not be the most important factor for myocardialization to proceed. Reduced myocardialization may reflect reduced levels of an inductive factor or a reduced number of one or more cell subpopulations that contribute to the heart cushions [61]–[67].

Because versican is an extracellular matrix molecule, the cellular responses reflected by the differentially expressed proteins are likely due to the cells interaction with an altered ECM. Periostin is an important molecule of the heart cushion extracellular matrix [68] and showed an altered distribution in Vcan^(tm1Zim)^ mutant cushions. Vcan induced changes in periostin distribution may effect the developing valves by altering the structural integrity of the ECM through altered interaction with other molecules such as tenascin-C, fibronectin, collagen and other proteoglycans [40], [69], [70]. Additionally, because it interacts directly with integrin, attachment-dependent signaling [71], [72] may be altered affecting cell migration and epithelial mesenchymal transition in the cushion primordia of the septa and valves. We identified by ITRAQ analysis changes in other proteins involved in extracellular matrix organization and cell response. These include serpinh1 (Hsp 47) that is required for normal collagen deposition [73]. Also, calreticulin required for collagen deposition and fibulin 5 needed for elastin assembly showed reduced abundance in the mutant heart by iTRAQ and are candidates for future investigation [74], [75]. Both of these last two ECM molecules are important for development and maintenance of the great arteries. In addition, changes in abundance were measured in ECM signal transduction proteins. Annexin A6 (reduced 75%) has been shown to directly bind versican and is a putative cell surface receptor for chondroitin sulfate proteoglycans [76]. This Ca++ regulatory protein is highly expressed in various cells of the heart and modulates EGFR stimulated Ca++ influx [77]. Inhibition of Annexin A6 expression by siRNA enhances EGF stimulated phosphorylation in the Ras/MAPK pathway [78], [79]. It is also interesting, but not yet verified independently, that changes were measured by iTRAQ in the proteins Fkbp1a and Rbpms that both regulate the ECM associated Smad pathway that may potentially play a role in the mutant phenotype. Also, the dilated right ventricular phenotype in the *Vcan* ^(tm1Zim)^ hearts correlated with protein abundance changes identified in the proteomic results. Among these, a protein that showed changes in abundance by the iTRAQ analysis was LIM domain binding 3 isoform b also known as cypher/ZASP. Cypher/ZASP is a member of the family of proteins containing a PDZ domain at their amino terminus and LIM domains at their COOH terminus. Cypher null mice display a severe form of congenital myopathy and die postnatally from functional failure in multiple striated muscles [80]. Mutations in the human homolog of cypher/ZASP have been associated with dilated cardiomyopathy and left ventricular non-compaction [81]–[84]. Reduction in the abundance of vimentin was also noted by iTRAQ in the *Vcan* ^(tm1Zim)^ and may be related to the observed lower mesenchyme cell density in the endocardial cushions. The versican V1 (exon 8 containing) splice form still present in the *Vcan* ^(tm1Zim)^ down regulates the expression of vimentin in NIH 3T3 cells [85]. Although vimentin expression is not normally abundant in adult cardiac myocytes, it is upregulated in regenerating [86] and embryonic myocytes during differentiation [87]. It should be noted that the ITRAQ proteomic results do not represent the entire proteome and that these data would need further validation and functional experiments to make a link with any candidate protein and the observed defects.

In conclusion, the defects in cardiac phenotype and alterations in protein abundance reported in this study show that the versican splice forms V1/V2 containing exon 7 are important for regulation of cardiac cushion size and the myocardial remodeling required for the proper integration of the outflow tract into the base of the heart.

## Supporting Information

File S1 **Supplemental iTRAQ report.**
(PDF)

File S2 **Supplemental GO file.**
(PDF)
