# Supplementary material for: Imbalanced Expression of Vcan mRNA Splice Form Proteins Alters Heart Morphology and Cellular Protein Profiles
Source: PLoS One. 2014 Feb 20;9(2):e89133. doi: 10.1371/journal.pone.0089133 (PMC3930639; doi:10.1371/journal.pone.0089133)
Supplement: File S1 — Supplemental iTRAQ report. (PDF) [file pone.0089133.s001.pdf]

iTRAQ Data Analysis Report

Burns et al., 2013

Krug, Mouse Heart IX 28

March 22, 2012

1 Introduction

This document summarizes an analysis of relative protein expression using iTRAQ. The reporter ion peak area measurements supplied by the ABI software are used to estimate treatment-dependent peptide and protein relative expression. Estimation is accomplished using a Bayesian approach with the model given below. The document includes a protein relative expression summary and a per-protein detailed analysis. The document is internally hyperlinked and linked externally to NCBI.

2 Experiment and Model Description

2.1 Experiment Design

The report summarizes data from one or more iTRAQ experiments addressing a common comparison. The experiment design, used in this analysis, is given in the table below.

	Experiment	Treatment	Channel	Sample
1	A	CTL	114	S1
2	A	NULL	115	S2
3	A	CTL	116	S1
4	A	NULL	117	S2

2.2 Input Files

Data for this analysis was extracted from the following tandem mass spectra (MSMS) summary files.

Experiment	MSMS Summary File
A	SCW_IX_28_MSMSSummary_70.csv

2.3 Statistical Model

The following statistical model was used to estimate the treatment-dependent effects.

LogIntensity ˜ Channel + Spectrum + Protein + Peptide + Protein:Treatment + Peptide:Treatment

3 Data Summary

The data supplied in the MSMS summary is filtered to remove unidentified proteins, contaminants, and peptides containing selected modifications. The following table sumarizes the data provided and used in the analysis.

	A	Combined
Supplied Spectra	26705	26705
Unidentified Spectra	21608	21608
Disallowed Modifications	0	0
Spectra from Contaminants	0	0
Missing Data	2	2
Low Confidence Spectra	0	0
Degenerate Peptides	0	0
Remaining Spectra		5095
Unique Proteins		940
Unique Peptides		2882
Model R^2		0.972

4 Protein Summary

Each protein identified in one or more of the MSMS summaries is listed below in decreasing order of expression change magnitude. The median and estimated credible interval for each protein is given to the left in the table. Proteins identified by a single peptide are listed in a separate table.

4.1 Identified Proteins

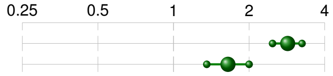

Peptides	Spectra	Coverage	Protein
6	7	0.182	annexin A5 [Mus musculus]
2	3	0.150	RNA polymerase II transcriptional coactivator [Mus musculus]

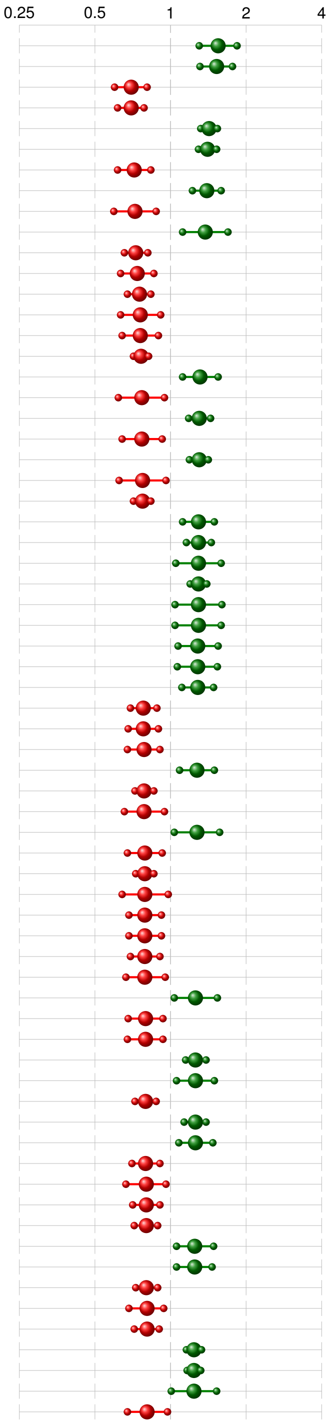

Peptides	Spectra	Coverage	Protein
3	4	0.108	serine (or cysteine) proteinase inhibitor, clade H, member 1 [Mus musculus]
4	6	0.139	Tu translation elongation factor, mitochondrial [Mus musculus]
4	6	0.5	thymosin, beta 4, X chromosome [Mus musculus]
5	12	0.442	hemoglobin, beta adult minor chain [Mus musculus]
12	44	NaN	glyceraldehyde-3-phosphate dehydrogenase [Mus musculus]
16	23	0.103	myosin heavy chain 10, non-muscle [Mus musculus]
4	6	NaN	PREDICTED: similar to Chromobox protein homolog 1 (Heterochromatin protein 1 homolog beta) (HP1 beta) (Modific
6	8	0.197	heterogeneous nuclear ribonucleoprotein K [Mus musculus]
2	3	0.125	RAS-related C3 botulinum substrate 1 [Mus musculus]
2	2	0.228	DJ-1 protein [Mus musculus]
9	13	0.57	stathmin 1 [Mus musculus]
4	6	0.351	myoglobin [Mus musculus]
4	43	NaN	hemoglobin alpha 1 chain [Mus musculus]
3	3	0.327	cystatin B [Mus musculus]
3	5	0.148	vacuolar protein sorting 29 [Mus musculus]
13	60	0.486	triosephosphate isomerase 1 [Mus musculus]
3	5	0.0366	phosphoribosylglycinamide formyltransferase [Mus musculus]
2	2	0.461	protein kinase inhibitor, alpha [Mus musculus]
10	16	NaN	PREDICTED: similar to 60 kDa heat shock protein, mitochondrial precursor (Hsp60) (60 kDa chaperonin) (CPN60) (H
2	5	0.318	thymosin, beta 10 [Mus musculus]
9	29	NaN	eukaryotic translation elongation factor 1 alpha 1 [Mus musculus]
2	2	0.235	neural precursor cell expressed, developmentally down-regulated gene 8 [Mus musculus]
12	47	0.707	peptidylprolyl isomerase A [Mus musculus]
5	6	0.105	dihydropyrimidinase-like 2 [Mus musculus]
6	13	0.220	protein disulfide isomerase-associated 6 [Mus musculus]
2	2	NaN	PREDICTED: similar to Splicing factor, arginine/serine-rich 3 (Pre-mRNA splicing factor SRP20) (X16 protein) [Mus m
18	34	0.235	eukaryotic translation elongation factor 2 [Mus musculus]
2	2	0.0631	fumarate hydratase 1 [Mus musculus]
2	2	0.0636	aldehyde dehydrogenase 1 family, member B1 [Mus musculus]
3	3	0.0604	heterogeneous nuclear ribonucleoprotein M [Mus musculus]
3	3	NaN	histidine rich calcium binding protein [Mus musculus]
5	6	NaN	t-complex protein 1 [Mus musculus]
7	11	NaN	nuclear autoantigenic sperm protein [Mus musculus]
4	8	0.243	prothymosin alpha [Mus musculus]
4	6	0.114	SET translocation [Mus musculus]
4	5	0.118	acetyl-Coenzyme A acetyltransferase 1 precursor [Mus musculus]
11	30	0.637	myosin, light polypeptide 3 [Mus musculus]
3	3	0.0332	ubiquitin specific protease 47 [Mus musculus]
2	2	0.0859	microtubule-associated protein, RP/EB family, member 2 [Mus musculus]
3	5	0.209	cell division cycle 42 homolog [Mus musculus]
14	26	0.260	calreticulin [Mus musculus]
2	2	0.0988	purine rich element binding protein B [Mus musculus]
4	6	NaN	RAN binding protein 1 [Mus musculus]
3	7	0.387	phosphohistidine phosphatase [Mus musculus]
4	10	0.244	ras homolog gene family, member A [Mus musculus]
2	5	0.312	cysteine-rich protein 1 (intestinal) [Mus musculus]
2	3	0.0652	TAR DNA binding protein isoform 1 [Mus musculus]
3	6	NaN	nuclease sensitive element binding protein 1 [Mus musculus]
3	6	0.2	thioredoxin 1 [Mus musculus]
10	21	0.553	peroxiredoxin 1 [Mus musculus]
3	4	0.144	electron transferring flavoprotein, alpha polypeptide [Mus musculus]
10	18	0.204	albumin 1 [Mus musculus]
7	19	0.186	isocitrate dehydrogenase 2 (NADP+), mitochondrial [Mus musculus]
4	5	0.109	isocitrate dehydrogenase 3 (NAD+) alpha [Mus musculus]
5	10	0.461	eukaryotic translation initiation factor 5A [Mus musculus]
3	3	NaN	Bcl2-associated athanogene 3 [Mus musculus]
7	9	0.0582	A kinase (PRKA) anchor protein (gravin) 12 [Mus musculus]
7	15	0.448	cysteine and glycine-rich protein 3 [Mus musculus]
4	4	0.131	PREDICTED: heterogeneous nuclear ribonucleoprotein A0 isoform 2 [Mus musculus]
4	4	NaN	chaperonin subunit 8 (theta) [Mus musculus]
8	21	0.417	Rho GDP dissociation inhibitor (GDI) alpha [Mus musculus]
2	8	0.205	troponin C, cardiac/slow skeletal [Mus musculus]
8	11	0.161	nexilin [Mus musculus]
16	60	0.415	tubulin, alpha 2 [Mus musculus]
17	106	0.493	tubulin, beta 5 [Mus musculus]
2	2	0.119	actin related protein 2/3 complex, subunit 4 [Mus musculus]
2	4	0.204	tubulin cofactor a [Mus musculus]

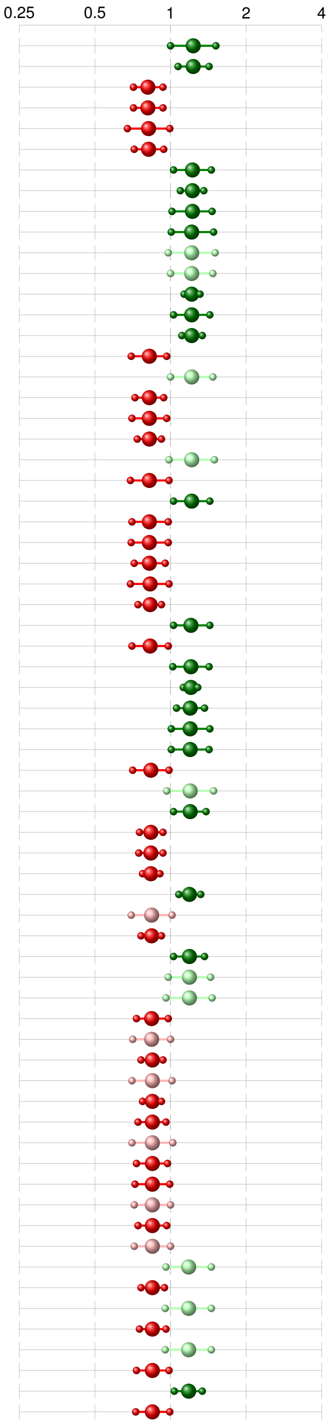

Peptides	Spectra	Coverage	Protein
2	2	0.0342	oxoglutarate dehydrogenase (lipoamide) [Mus musculus]
5	7	0.105	ATP synthase, H+ transporting, mitochondrial F1 complex, alpha subunit, isoform 1 [Mus musculus]
6	7	0.122	adenosine kinase [Mus musculus]
6	7	0.0985	A kinase (PRKA) anchor protein 2 isoform 1 [Mus musculus]
2	3	0.0758	protein phosphatase 1, catalytic subunit, alpha [Mus musculus]
5	8	0.169	transaldolase 1 [Mus musculus]
2	5	0.0524	Rho GTPase activating protein 1 [Mus musculus]
9	14	0.207	dihydropyrimidinase-like 3 [Mus musculus]
3	3	0.138	succinate-CoA ligase, GDP-forming, alpha subunit [Mus musculus]
2	3	0.0433	guanine monphosphate synthetase [Mus musculus]
2	2	0.131	ubiquitin-conjugating enzyme E2M [Mus musculus]
2	3	0.168	RNA binding protein gene with multiple splicing [Mus musculus]
18	33	0.276	aconitase 2, mitochondrial [Mus musculus]
4	4	NaN	CAP, adenylate cyclase-associated protein 1 [Mus musculus]
11	23	0.349	heterogeneous nuclear ribonucleoprotein A2/B1 isoform 2 [Mus musculus]
3	5	NaN	PREDICTED: similar to PDZ and LIM domain protein 1 (Elfin) (LIM domain protein CLP-36) (C-terminal LIM domain p
2	3	0.0160	fatty acid synthase [Mus musculus]
6	8	0.155	FK506 binding protein 4 [Mus musculus]
3	6	NaN	PREDICTED: similar to Prostaglandin E synthase 3 (Cytosolic prostaglandin E2 synthase) (cPGES) (Telomerase-bin
6	19	0.407	diazepam binding inhibitor isoform 1 [Mus musculus]
2	2	0.0584	PREDICTED: similar to Probable ATP-dependent RNA helicase DDX48 (DEAD box protein 48) (Eukaryotic initiation f
3	4	0.114	acyl-Coenzyme A dehydrogenase, short chain [Mus musculus]
4	4	0.101	CAP, adenylate cyclase-associated protein, 2 [Mus musculus]
4	4	0.0806	protein kinase C substrate 80K-H [Mus musculus]
3	4	0.246	programmed cell death 5 [Mus musculus]
4	7	0.149	myristoylated alanine rich protein kinase C substrate [Mus musculus]
3	4	0.141	tropomyosin 3, gamma [Mus musculus]
8	14	0.399	LIM only protein HLP [Mus musculus]
3	5	0.087	isocitrate dehydrogenase 1 (NADP+), soluble [Mus musculus]
3	5	0.182	3-monooxgenase/tryptophan 5-monooxygenase activation protein, gamma polypeptide [Mus musculus]
4	4	0.0256	talin 1 [Mus musculus]
24	55	0.542	pyruvate kinase 3 [Mus musculus]
6	9	NaN	PREDICTED: similar to electron transferring flavoprotein, beta polypeptide [Mus musculus]
2	6	0.0310	tubulin, alpha 1 [Mus musculus]
3	4	0.0758	chaperonin subunit 5 (epsilon) [Mus musculus]
3	5	0.415	fatty acid binding protein 5, epidermal [Mus musculus]
2	2	NaN	myosin, heavy polypeptide 9, non-muscle isoform 1 [Mus musculus]
3	9	0.0944	tubulin, beta, 2 [Mus musculus]
10	13	0.089	nestin [Mus musculus]
9	13	0.61	phosphatidylethanolamine binding protein 1 [Mus musculus]
15	32	0.279	alpha fetoprotein [Mus musculus]
9	17	0.394	PREDICTED: similar to High mobility group protein 1 (HMG-1) (High mobility group protein B1) (Amphoterin) (Hepar
3	3	0.118	retinoblastoma binding protein 4 [Mus musculus]
9	25	NaN	heat shock protein 1 [Mus musculus]
6	6	0.138	HLA-B-associated transcript 1A [Mus musculus]
2	3	NaN	eukaryotic translation elongation factor 1 gamma [Mus musculus]
2	2	0.0414	programmed cell death 6 interacting protein [Mus musculus]
4	7	0.103	fibulin 5 [Mus musculus]
3	4	0.152	hepatoma-derived growth factor [Mus musculus]
9	19	0.361	tyrosine 3-monooxygenase/tryptophan 5-monooxygenase activation protein, epsilon polypeptide [Mus musculus]
3	3	0.089	RAD23b homolog [Mus musculus]
10	35	0.355	tyrosine 3-monooxygenase/tryptophan 5-monooxygenase activation protein, zeta polypeptide [Mus musculus]
5	9	0.170	suppression of tumorigenicity 13 [Mus musculus]
3	3	0.229	adenylate kinase 1 [Mus musculus]
4	8	0.181	calumenin isoform 1 [Mus musculus]
3	6	0.194	endoplasmic reticulum protein ERp19 [Mus musculus]
2	6	0.148	glutathione S-transferase, pi 1 [Mus musculus]
5	9	0.44	cofilin 2, muscle [Mus musculus]
3	5	0.103	guanosine diphosphate (GDP) dissociation inhibitor 1 [Mus musculus]
2	2	0.000926	titin isoform N2-A [Mus musculus]
8	18	0.514	profilin 1 [Mus musculus]
2	2	0.0601	melanoma antigen family D, 2 [Mus musculus]
7	10	NaN	myomesin 1 [Mus musculus]
2	2	NaN	PREDICTED: SEC31-like 1 [Mus musculus]
4	6	0.169	tropomyosin 4 [Mus musculus]
8	8	0.0467	microtubule-associated protein 1 B [Mus musculus]
5	5	0.222	aldo-keto reductase family 1, member A4 (aldehyde reductase) [Mus musculus]

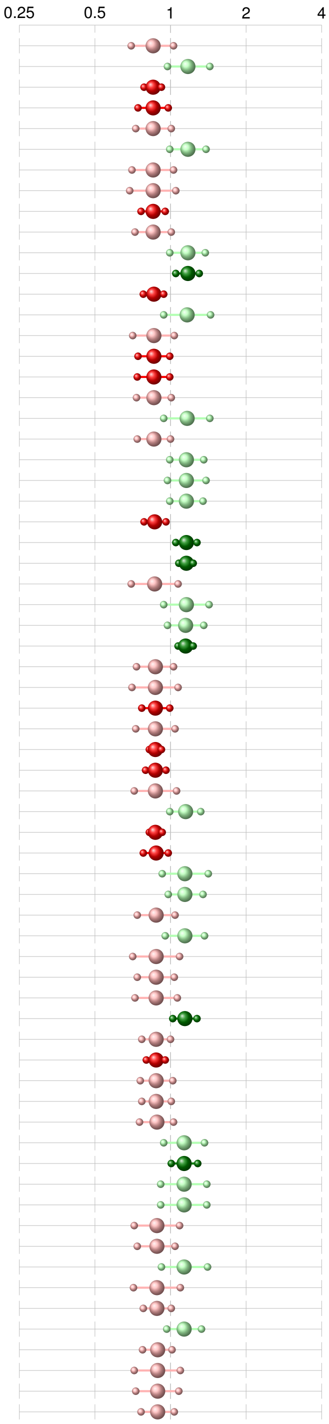

Peptides	Spectra	Coverage	Protein
2	3	0.169	myotrophin [Mus musculus]
2	3	0.0277	myosin light chain kinase [Mus musculus]
16	28	NaN	PREDICTED: similar to sorbin and SH3 domain containing 2 isoform 1 isoform 21 [Mus musculus]
5	8	0.42	cysteine and glycine-rich protein 1 [Mus musculus]
3	5	0.145	esterase D/formylglutathione hydrolase [Mus musculus]
4	4	0.169	AHA1, activator of heat shock 90kDa protein ATPase homolog 1 [Mus musculus]
2	4	NaN	PREDICTED: similar to Retinol-binding protein I, cellular (Cellular retinol-binding protein) (CRBP) (mCRBPI) [Mus mu
2	2	NaN	PREDICTED: similar to EF hand domain containing 2 isoform 2 [Mus musculus]
8	13	0.349	p47 protein [Mus musculus]
4	4	0.307	parathymosin [Mus musculus]
4	4	0.088	cytidine 5'-triphosphate synthase [Mus musculus]
7	17	0.193	ATP synthase, H+ transporting mitochondrial F1 complex, beta subunit [Mus musculus]
8	28	0.446	myosin, light polypeptide 4 [Mus musculus]
2	2	0.194	histone 1, H4k [Mus musculus]
2	3	0.571	cytochrome c oxidase subunit XVII assembly protein homolog [Mus musculus]
5	6	NaN	fascin homolog 1, actin bundling protein (Strongylocentrotus) purpuratus) [Mus musculus]
4	6	0.181	small glutamine-rich tetratricopeptide repeat (TPR) containing protein [Mus musculus]
4	5	0.0788	annexin A6 [Mus musculus]
2	2	0.0354	TNF receptor-associated protein 1 [Mus musculus]
3	7	0.647	small muscle protein, X-linked [Mus musculus]
4	5	0.107	alpha isoform of regulatory subunit A, protein phosphatase 2 [Mus musculus]
3	4	0.123	ubiquitin-like 1 (sentrin) activating enzyme subunit 1 [Mus musculus]
4	6	0.204	dodecenoyl-Coenzyme A delta isomerase (3,2 trans-enoyl-Coenyme A isomerase) [Mus musculus]
9	19	0.282	tyrosine 3-monooxygenase/tryptophan 5-monooxygenase activation protein, theta polypeptide [Mus musculus]
11	17	0.199	valosin containing protein [Mus musculus]
20	58	0.537	hypothetical protein LOC433182 [Mus musculus]
2	2	0.132	peptidylprolyl isomerase C [Mus musculus]
2	2	0.151	ATPase inhibitory factor 1 [Mus musculus]
4	4	0.428	ubiquitin-conjugating enzyme E2N [Mus musculus]
16	49	NaN	PREDICTED: similar to Phosphoglycerate mutase 1 (Phosphoglycerate mutase isozyme B) (PGAM-B) (BPG-depende
4	4	NaN	clathrin, light polypeptide (Lca) [Mus musculus]
2	2	0.159	fatty acid binding protein 4, adipocyte [Mus musculus]
5	9	0.339	PREDICTED: similar to myosin, light polypeptide 6, alkali, smooth muscle and non-muscle [Mus musculus]
3	3	NaN	PREDICTED: similar to Calpastatin (Calpain inhibitor) [Mus musculus]
31	67	0.651	tropomyosin 1, alpha [Mus musculus]
9	28	0.215	glucose phosphate isomerase 1 [Mus musculus]
2	3	0.163	nuclear, casein kinase and cyclin-dependent kinase substrate [Mus musculus]
5	7	0.249	proliferating cell nuclear antigen [Mus musculus]
25	70	0.519	vimentin [Mus musculus]
6	15	NaN	myosin, light polypeptide 2, regulatory, cardiac, slow [Mus musculus]
2	2	0.0416	tryptophanyl-tRNA synthetase [Mus musculus]
4	5	NaN	PREDICTED: similar to cardiomyopathy associated 4 [Mus musculus]
3	4	0.0857	nucleobindin 1 [Mus musculus]
3	3	0.0115	plectin 1 isoform 4 [Mus musculus]
2	2	0.0742	serum deprivation response [Mus musculus]
3	4	0.0735	eukaryotic translation initiation factor 3, subunit 9 [Mus musculus]
2	3	0.0514	far upstream element (FUSE) binding protein 1 [Mus musculus]
8	12	NaN	heat shock protein 4 [Mus musculus]
6	8	0.302	splicing factor, arginine/serine-rich 1 (ASF/SF2) [Mus musculus]
14	20	0.236	nucleolin [Mus musculus]
4	7	0.186	LIM and SH3 protein 1 [Mus musculus]
5	8	0.059	alpha glucosidase 2 alpha neutral subunit [Mus musculus]
4	5	0.08	thimet oligopeptidase 1 [Mus musculus]
2	4	0.133	capping protein (actin filament) muscle Z-line, alpha 1 [Mus musculus]
6	11	0.24	heterogeneous nuclear ribonucleoprotein A3 isoform b [Mus musculus]
2	2	0.0427	glucan (1,4-alpha-), branching enzyme 1 [Mus musculus]
2	2	NaN	PREDICTED: H3 histone, family 2 [Mus musculus]
2	2	0.096	RIKEN cDNA 0610040D20 [Mus musculus]
3	4	0.185	carnitine deficiency-associated gene expressed in ventricle 3 isoform CDV3B [Mus musculus]
2	2	0.0284	HGF-regulated tyrosine kinase substrate [Mus musculus]
2	2	0.104	APAF1 interacting protein [Mus musculus]
5	9	0.187	melanocyte proliferating gene 1 [Mus musculus]
3	5	0.0895	tubulin, beta 6 [Mus musculus]
5	11	0.389	FK506 binding protein 1a [Mus musculus]
2	2	0.116	hypothetical protein LOC69099 [Mus musculus]
2	3	0.0957	v-crk sarcoma virus CT10 oncogene homolog (avian)-like [Mus musculus]
5	5	0.367	hemoglobin Z, beta-like embryonic chain [Mus musculus]

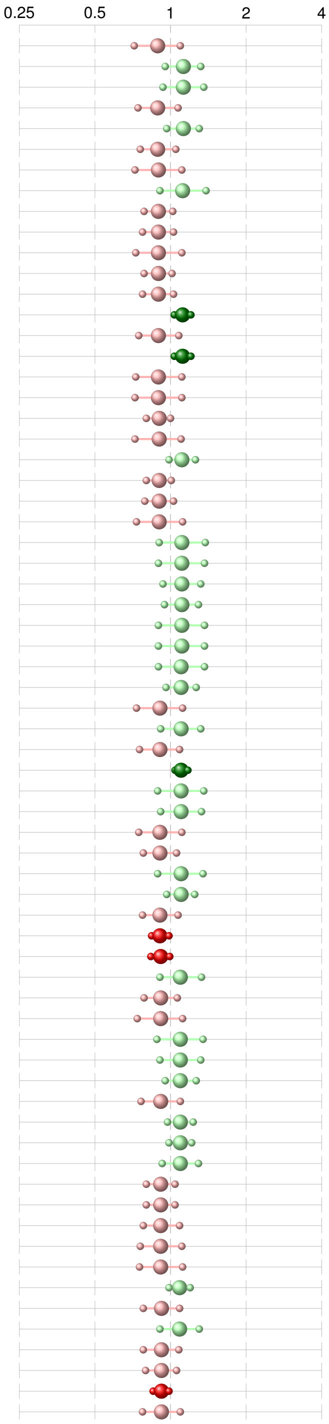

Peptides	Spectra	Coverage	Protein
2	2	0.0804	chromatin modifying protein 4B [Mus musculus]
4	4	0.152	protein kinase, cAMP dependent regulatory, type I, alpha [Mus musculus]
3	3	0.065	phosphofructokinase, platelet [Mus musculus]
3	3	0.170	ubiquitin carboxy-terminal hydrolase L1 [Mus musculus]
4	6	0.273	thioredoxin-like 1 [Mus musculus]
3	5	0.386	SH3-binding domain glutamic acid-rich protein like [Mus musculus]
2	2	NaN	PREDICTED: similar to RNA binding motif protein 24 isoform 3 [Mus musculus]
2	2	NaN	structure specific recognition protein 1 [Mus musculus]
6	8	NaN	PREDICTED: similar to Bifunctional purine biosynthesis protein PURH isoform 2 [Mus musculus]
6	6	0.0344	translocated promoter region protein [Mus musculus]
2	2	0.0662	guanine nucleotide binding protein (G protein), beta polypeptide 2 like 1 [Mus musculus]
6	8	0.113	LIM domain binding 3 isoform b [Mus musculus]
6	6	0.166	autoantigen La [Mus musculus]
20	27	NaN	PREDICTED: similar to Filamin-A (Alpha-filamin) (Filamin-1) (Endothelial actin-binding protein) (Actin-binding protei
3	3	0.0805	platelet-activating factor acetylhydrolase, isoform 1b, beta1 subunit [Mus musculus]
19	25	0.155	clathrin, heavy polypeptide (Hc) [Mus musculus]
2	2	0.125	nucleoside-diphosphate kinase 1 [Mus musculus]
2	2	0.156	prefoldin 2 [Mus musculus]
8	13	0.117	actinin alpha 4 [Mus musculus]
2	2	0.139	core binding factor beta [Mus musculus]
5	13	0.17	eukaryotic translation initiation factor 4A1 [Mus musculus]
7	14	0.217	heterogeneous nuclear ribonucleoprotein F [Mus musculus]
5	10	0.471	heat shock protein 1 (chaperonin 10) [Mus musculus]
2	2	0.0674	N-myc downstream regulated gene 2 [Mus musculus]
2	2	NaN	spectrin beta 2 isoform 1 [Mus musculus]
2	2	NaN	PREDICTED: similar to PDZ and LIM domain protein 5 (Enigma homolog) (Enigma-like PDZ and LIM domains protein
3	4	0.0356	muscle glycogen phosphorylase [Mus musculus]
4	5	NaN	PREDICTED: similar to laminin receptor 1 (ribosomal protein SA) isoform 2 [Mus musculus]
2	2	0.0976	isochorismatase domain containing 1 [Mus musculus]
2	2	0.0328	ribonucleotide reductase M1 [Mus musculus]
2	2	0.0334	minichromosome maintenance protein 7 [Mus musculus]
3	15	0.133	actin, gamma, cytoplasmic 1 [Mus musculus]
2	2	0.0924	sorbitol dehydrogenase 1 [Mus musculus]
3	3	NaN	PREDICTED: similar to High mobility group protein 2 (HMG-2) [Mus musculus]
2	4	0.0836	cyclin-dependent kinase inhibitor 1C (P57) [Mus musculus]
30	51	0.2	myosin, heavy polypeptide 7, cardiac muscle, beta [Mus musculus]
2	2	0.0983	poly(rC) binding protein 1 [Mus musculus]
2	4	0.0296	hexokinase 1 [Mus musculus]
2	3	0.131	biliverdin reductase B (flavin reductase (NADPH)) [Mus musculus]
3	7	0.236	cellular nucleic acid binding protein 1 [Mus musculus]
2	2	0.0215	nardilysin, N-arginine dibasic convertase, NRD convertase 1 [Mus musculus]
6	8	0.0728	aminopeptidase puromycin sensitive [Mus musculus]
3	5	0.0674	X-prolyl aminopeptidase (aminopeptidase P) 1, soluble [Mus musculus]
14	35	0.503	malate dehydrogenase 2, NAD (mitochondrial) [Mus musculus]
13	29	0.379	aldolase 1, A isoform [Mus musculus]
2	3	NaN	heme binding protein 1 [Mus musculus]
4	6	0.0664	KH-type splicing regulatory protein [Mus musculus]
2	2	0.085	RCSD domain containing 1 isoform a [Mus musculus]
2	2	0.0424	sorting nexin 2 [Mus musculus]
3	3	0.0478	NOL1/NOP2/Sun domain family 2 [Mus musculus]
3	9	0.0809	tubulin, beta [Mus musculus]
3	3	NaN	ubiquitin carboxyl-terminal esterase L3 (ubiquitin thiolesterase) [Mus musculus]
7	13	0.149	phosphoglucomutase 2 [Mus musculus]
12	13	NaN	spectrin beta 2 isoform 2 [Mus musculus]
4	4	0.123	S-adenosylhomocysteine hydrolase [Mus musculus]
6	8	NaN	PREDICTED: similar to ring finger protein 29 (predicted) isoform 1 [Mus musculus]
6	8	0.225	aldo-keto reductase family 1, member B3 (aldose reductase) [Mus musculus]
3	5	0.42	transcription elongation factor B (SIII), polypeptide 1 [Mus musculus]
2	3	0.157	lymphocyte antigen 6 complex, locus E ligand [Mus musculus]
2	3	0.0722	inositol (myo)-1(or 4)-monophosphatase 1 [Mus musculus]
8	21	NaN	PREDICTED: similar to GDP dissociation inhibitor 2 isoform 3 [Mus musculus]
4	4	NaN	laminin, gamma 1 [Mus musculus]
3	3	0.0657	SET and MYND domain containing 1 [Mus musculus]
3	5	0.0974	acetyl-Coenzyme A dehydrogenase, medium chain [Mus musculus]
5	7	0.27	troponin I, cardiac [Mus musculus]
22	30	0.52	desmin [Mus musculus]
3	4	0.118	drebrin-like [Mus musculus]

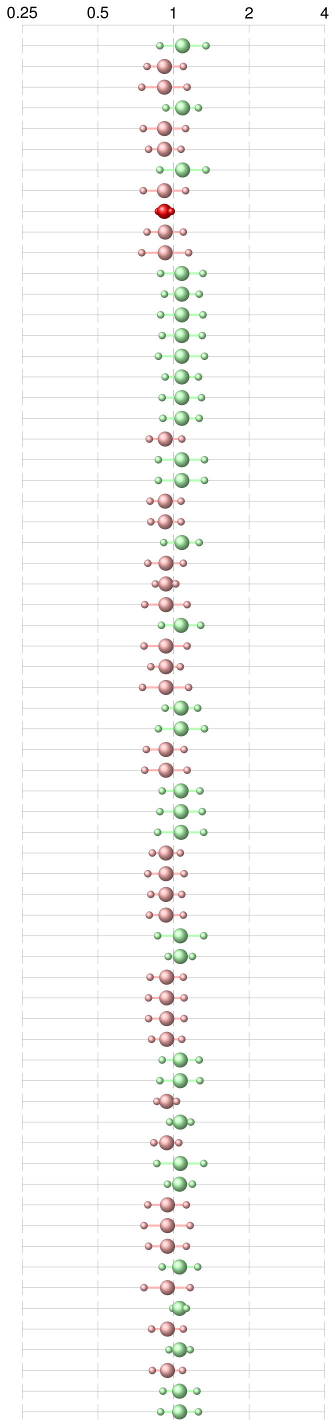

Peptides	Spectra	Coverage	Protein
2	2	NaN	PREDICTED: similar to H2A histone family, member Z isoform 2 [Mus musculus]
3	5	0.161	Williams-Beuren syndrome chromosome region 1 homolog [Mus musculus]
2	2	0.0196	Na+/K+ -ATPase alpha 1 subunit [Mus musculus]
3	7	0.0627	transglutaminase 2, C polypeptide [Mus musculus]
2	3	0.2	PREDICTED: similar to UPF0315 protein [Mus musculus]
5	5	NaN	radixin [Mus musculus]
2	2	0.134	GrpE-like 1, mitochondrial [Mus musculus]
2	3	0.0735	acidic nuclear phosphoprotein 32 family, member B [Mus musculus]
22	62	NaN	PREDICTED: similar to heat shock protein 8 [Mus musculus]
2	9	0.174	macrophage migration inhibitory factor [Mus musculus]
2	2	NaN	four and a half LIM domains 1 [Mus musculus]
2	3	NaN	PREDICTED: phosphogluconate dehydrogenase isoform 4 [Mus musculus]
4	5	NaN	PREDICTED: similar to Filamin-B (FLN-B) (Beta-filamin) (Actin-binding-like protein) (ABP-280-like protein) isoform 7
2	3	0.296	high mobility group AT-hook 2 [Mus musculus]
3	3	0.0564	synaptopodin 2-like [Mus musculus]
2	2	0.0554	cell division cycle 37 homolog [Mus musculus]
5	5	0.155	alcohol dehydrogenase 5 (class III), chi polypeptide [Mus musculus]
3	3	0.106	PREDICTED: similar to eukaryotic translation initiation factor 4E [Mus musculus]
4	4	NaN	myosin binding protein C, cardiac [Mus musculus]
4	6	0.154	nuclear distribution gene C homolog [Mus musculus]
2	2	0.11	RAB10, member RAS oncogene family [Mus musculus]
2	2	0.0931	glycogenin 1 [Mus musculus]
4	7	0.203	tyrosine 3-monooxygenase/tryptophan 5-monooxygenase activation protein, eta polypeptide [Mus musculus]
4	8	0.0614	nucleosome assembly protein 1-like 1 [Mus musculus]
3	5	0.0445	karyopherin (importin) beta 1 [Mus musculus]
2	8	0.183	PREDICTED: similar to Heat-shock protein beta-7 (HspB7) (Cardiovascular heat shock protein) (cvHsp) (Heat shock
11	17	0.214	transferrin [Mus musculus]
2	3	0.0777	protein phosphatase 2a, catalytic subunit, beta isoform [Mus musculus]
3	3	0.197	proteasome (prosome, macropain) 28 subunit, alpha [Mus musculus]
2	3	0.0287	epidermal growth factor receptor pathway substrate 15, related [Mus musculus]
5	8	NaN	microtubule-associated protein 4 [Mus musculus]
2	2	0.0708	reticulocalbin 1 [Mus musculus]
4	6	0.235	splicing factor, arginine/serine-rich 7 [Mus musculus]
2	2	0.0277	adaptor-related protein complex 2, beta 1 subunit isoform b [Mus musculus]
3	4	0.09	opioid growth factor receptor [Mus musculus]
2	3	0.0513	serine/threonine kinase receptor associated protein [Mus musculus]
3	4	0.131	hypothetical protein LOC67078 [Mus musculus]
2	3	0.0376	hypothetical protein LOC223723 [Mus musculus]
2	2	0.0362	alanyl-tRNA synthetase [Mus musculus]
6	9	0.176	zyxin [Mus musculus]
3	5	0.154	chloride intracellular channel 1 [Mus musculus]
5	7	0.235	hypothetical protein LOC67201 [Mus musculus]
4	5	0.286	peroxiredoxin 5 precursor [Mus musculus]
2	2	NaN	PREDICTED: similar to phosphoribosylformylglycinamidine synthase [Mus musculus]
9	12	0.237	lactate dehydrogenase 2, B chain [Mus musculus]
3	7	NaN	PREDICTED: similar to nascent polypeptide-associated complex alpha polypeptide [Mus musculus]
3	6	0.215	tumor protein, translationally-controlled 1 [Mus musculus]
4	4	0.06	tripartite motif protein 28 [Mus musculus]
5	7	0.124	LIM domain containing preferred translocation partner in lipoma [Mus musculus]
3	4	0.150	capping protein (actin filament) muscle Z-line, alpha 2 [Mus musculus]
3	3	0.128	SGT1, suppressor of G2 allele of SKP1 [Mus musculus]
13	20	0.199	moesin [Mus musculus]
9	18	0.138	neural precursor cell expressed, developmentally down-regulated gene 4 [Mus musculus]
4	23	0.345	hemoglobin X, alpha-like embryonic chain in Hba complex [Mus musculus]
2	2	0.0287	isoleucine-tRNA synthetase 2, mitochondrial [Mus musculus]
6	15	0.173	creatine kinase, brain [Mus musculus]
3	4	0.179	HN1-like protein [Mus musculus]
2	2	0.208	RNA binding motif protein 3 [Mus musculus]
2	5	0.0668	heterogeneous nuclear ribonucleoprotein H1 [Mus musculus]
3	5	0.174	transgelin [Mus musculus]
2	2	NaN	NudC domain containing 2 [Mus musculus]
30	47	NaN	PREDICTED: similar to Filamin-C (Gamma-filamin) (Filamin-2) (Protein FLNc) (Actin-binding-like protein) (ABP-L) (A
4	7	0.115	thioredoxin domain containing 5 [Mus musculus]
10	16	0.355	lactate dehydrogenase 1, A chain [Mus musculus]
5	9	0.378	lectin, galactose binding, soluble 1 [Mus musculus]
4	5	0.0237	myosin, heavy polypeptide 6, cardiac muscle, alpha [Mus musculus]
3	4	0.128	glyoxylate reductase/hydroxypyruvate reductase [Mus musculus]

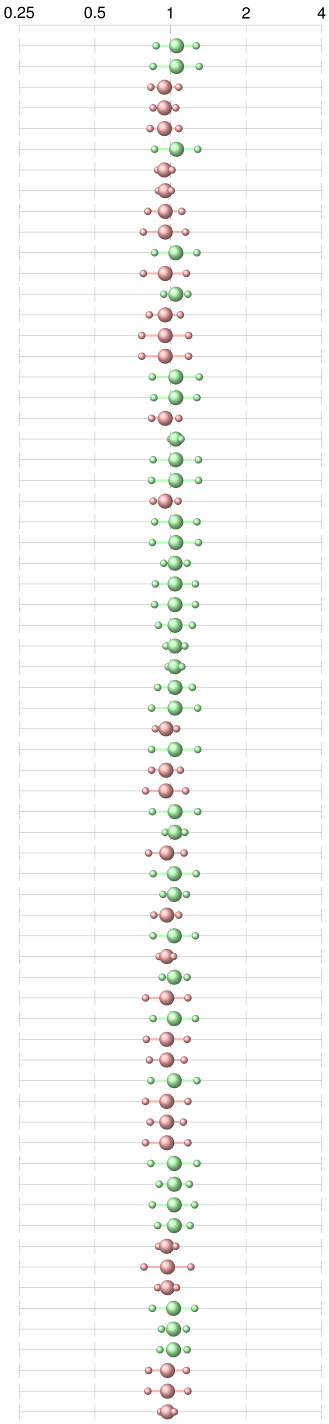

Peptides	Spectra	Coverage	Protein
3	3	NaN	PREDICTED: similar to palladin isoform 5 [Mus musculus]
2	2	NaN	peroxiredoxin 2 [Mus musculus]
6	9	0.167	acetyl-Coenzyme A dehydrogenase, long-chain [Mus musculus]
8	15	0.195	stress-induced phosphoprotein 1 [Mus musculus]
4	11	0.532	histidine triad nucleotide binding protein 1 [Mus musculus]
2	3	0.154	crystallin, alpha B [Mus musculus]
20	48	NaN	protein disulfide isomerase associated 3 [Mus musculus]
29	57	0.475	heat shock 70kD protein 5 (glucose-regulated protein) [Mus musculus]
3	7	0.269	heat shock protein 2 [Mus musculus]
2	3	0.0361	EIB-55kDa associated protein 5 isoform 1 [Mus musculus]
2	3	0.076	methionine adenosyltransferase II, alpha [Mus musculus]
2	3	0.046	acylpeptide hydrolase [Mus musculus]
6	18	0.5	nucleoside-diphosphate kinase 2 [Mus musculus]
4	7	0.191	tyrosine 3-monooxygenase/tryptophan 5-monooxygenase activation protein, beta polypeptide [Mus musculus]
2	2	0.0675	ELAV (embryonic lethal, abnormal vision, Drosophila)-like 1 (Hu antigen R) [Mus musculus]
2	2	0.0414	peripherin 1 [Mus musculus]
2	2	0.0240	G elongation factor 1 [Mus musculus]
2	3	0.0572	mannose-6-phosphate receptor binding protein 1 [Mus musculus]
7	9	0.220	glutamate oxaloacetate transaminase 1, soluble [Mus musculus]
34	102	0.399	heat shock protein 1, beta [Mus musculus]
2	2	0.0213	upstream of NRAS [Mus musculus]
2	2	0.198	histone 2, H2bb [Mus musculus]
8	13	0.321	troponin I, skeletal, slow 1 [Mus musculus]
2	3	0.0424	eukaryotic translation elongation factor 1 delta isoform a [Mus musculus]
2	2	0.0258	O-linked N-acetylglucosamine transferase [Mus musculus]
8	14	0.272	glutamate oxaloacetate transaminase 2, mitochondrial [Mus musculus]
3	3	0.110	polymerase I and transcript release factor [Mus musculus]
3	3	0.099	enolase 3, beta muscle [Mus musculus]
4	5	0.081	mitochondrial phosphoenolpyruvate carboxykinase 2 [Mus musculus]
14	25	0.413	troponin T2, cardiac [Mus musculus]
15	94	0.403	actin, alpha, cardiac [Mus musculus]
4	5	0.416	ubiquitin-conjugating enzyme E2L 3 [Mus musculus]
2	2	0.0665	casein kinase II, alpha 1 polypeptide [Mus musculus]
12	19	0.177	vinculin [Mus musculus]
2	2	0.0549	cytosolic acyl-CoA thioesterase 1 [Mus musculus]
6	8	0.202	creatine kinase, muscle [Mus musculus]
2	4	0.107	endoplasmic reticulum protein ERp29 precursor [Mus musculus]
2	2	0.0732	cathepsin D [Mus musculus]
10	42	0.762	hemoglobin, beta adult major chain [Mus musculus]
4	4	NaN	PREDICTED: similar to Cytochrome c, somatic [Mus musculus]
2	3	0.129	ubiquitin-conjugating enzyme E2 variant 1 [Mus musculus]
9	14	0.368	phosphoglycerate mutase 2 [Mus musculus]
7	11	0.452	transgelin 2 [Mus musculus]
2	3	0.159	brain abundant, membrane attached signal protein 1 [Mus musculus]
29	36	NaN	PREDICTED: spectrin alpha 2 isoform 17 [Mus musculus]
6	14	NaN	PREDICTED: similar to Peroxiredoxin-2 (Thioredoxin peroxidase 1) (Thioredoxin-dependent peroxide reductase 1) (T
2	3	0.0704	tropomyosin 2, beta [Mus musculus]
2	3	0.0293	PREDICTED: similar to p30 DBC protein [Mus musculus]
3	3	0.127	methylthioadenosine phosphorylase [Mus musculus]
4	5	0.133	apolipoprotein A-I [Mus musculus]
2	2	0.0363	proteasome 26S non-ATPase subunit 2 [Mus musculus]
2	3	NaN	proteasome 26S non-ATPase subunit 9 [Mus musculus]
5	5	0.106	arginyl aminopeptidase (aminopeptidase B) [Mus musculus]
2	3	0.232	cyclic AMP phosphoprotein, 19kD [Mus musculus]
2	2	NaN	caspase 6 [Mus musculus]
5	7	0.093	prolyl endopeptidase [Mus musculus]
3	3	0.0455	ubiquitin-like 1 (sentrin) activating enzyme E1B [Mus musculus]
4	6	0.212	PDZ and LIM domain 3 [Mus musculus]
10	46	0.59	cofilin 1, non-muscle [Mus musculus]
2	2	0.0144	latent transforming growth factor beta binding protein 4 [Mus musculus]
13	21	0.263	prolyl 4-hydroxylase, beta polypeptide [Mus musculus]
2	3	0.0686	capping protein (actin filament) muscle Z-line, beta isoform a [Mus musculus]
7	12	0.147	glycyl-tRNA synthetase [Mus musculus]
6	11	0.131	nucleosome assembly protein 1-like 4 [Mus musculus]
3	4	0.0637	dipeptidyl peptidase III [Mus musculus]
3	3	0.0404	damage specific DNA binding protein 1 [Mus musculus]
19	64	NaN	PREDICTED: similar to phosphoglycerate kinase 1 [Mus musculus]

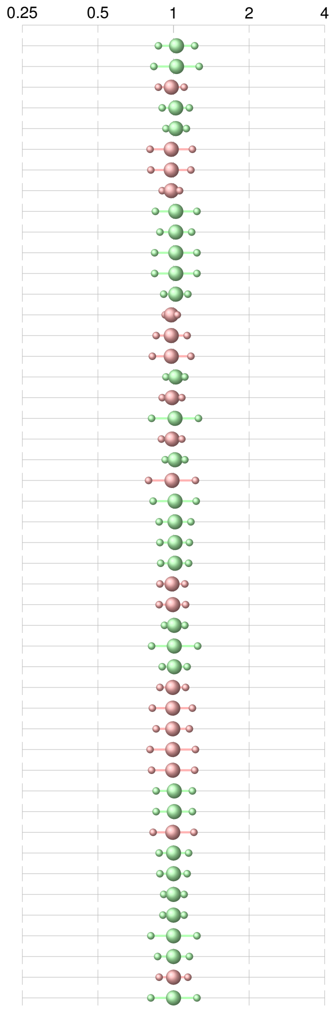

Peptides	Spectra	Coverage	Protein
3	5	NaN	PREDICTED: similar to chromobox homolog 3 [Mus musculus]
2	2	0.0284	fibulin 1 [Mus musculus]
5	17	NaN	PREDICTED: similar to Hemoglobin epsilon-Y2 subunit (Hemoglobin epsilon-Y2 chain) (Epsilon-Y2-globin) isoform :
5	11	0.29	peroxiredoxin 6 [Mus musculus]
13	18	0.177	actinin, alpha 1 [Mus musculus]
2	3	0.0853	clathrin, light polypeptide (Lcb) [Mus musculus]
2	4	0.107	pyrophosphatase [Mus musculus]
15	24	0.197	ubiquitin-activating enzyme E1, Chr X [Mus musculus]
2	3	0.0453	DIP13 alpha [Mus musculus]
4	7	0.0995	transketolase [Mus musculus]
2	3	0.0596	spermidine synthase [Mus musculus]
2	3	NaN	myosin, light polypeptide 7, regulatory [Mus musculus]
8	11	0.167	WD repeat domain 1 [Mus musculus]
27	70	NaN	actinin alpha 2 [Mus musculus]
4	8	NaN	PREDICTED: similar to ubiquitin A-52 residue ribosomal protein fusion product 1 [Mus musculus]
3	4	0.326	dynein, cytoplasmic, light peptide [Mus musculus]
12	26	0.175	heat shock protein 1, alpha [Mus musculus]
15	18	0.192	tumor rejection antigen gp96 [Mus musculus]
2	2	0.0392	basic transcription factor 3 [Mus musculus]
9	26	0.35	malate dehydrogenase 1, NAD (soluble) [Mus musculus]
13	19	0.249	heat shock protein 9A [Mus musculus]
2	2	0.0401	coronin, actin binding protein 1C [Mus musculus]
2	3	0.147	glyoxalase 1 [Mus musculus]
4	8	0.164	nucleophosmin 1 [Mus musculus]
7	7	0.108	ubiquitin specific protease 5 (isopeptidase T) [Mus musculus]
5	9	NaN	PREDICTED: similar to OTU domain, ubiquitin aldehyde binding 1 isoform 3 [Mus musculus]
6	16	NaN	heterogeneous nuclear ribonucleoprotein D [Mus musculus]
7	10	0.205	ornithine aminotransferase [Mus musculus]
11	22	0.394	RAN, member RAS oncogene family [Mus musculus]
2	2	0.0742	thioredoxin-like 2 [Mus musculus]
6	15	0.253	heterogeneous nuclear ribonucleoprotein A/B [Mus musculus]
8	10	NaN	PREDICTED: similar to Leukotriene A-4 hydrolase (LTA-4 hydrolase) (Leukotriene A(4) hydrolase) [Mus musculus]
3	3	0.127	protein kinase, cAMP dependent regulatory, type II alpha [Mus musculus]
5	5	0.119	cortactin [Mus musculus]
2	2	0.145	cysteine and glycine-rich protein 2 [Mus musculus]
2	3	0.0792	thyroid receptor-interacting protein 6 [Mus musculus]
4	4	0.0739	poly A binding protein, cytoplasmic 1 [Mus musculus]
3	5	0.170	transmembrane protein 4 [Mus musculus]
3	3	0.104	GTP-binding protein PTD004 isoform a [Mus musculus]
6	7	0.104	gelsolin [Mus musculus]
5	13	0.161	heterogeneous nuclear ribonucleoprotein A1 isoform b [Mus musculus]
9	22	0.491	destrin [Mus musculus]
12	16	NaN	PREDICTED: cardiomyopathy associated 1 isoform 2 [Mus musculus]
2	2	NaN	PREDICTED: similar to Putative eukaryotic translation initiation factor 3 subunit (eIF-3) isoform 12 [Mus musculus]
5	6	0.272	6-phosphogluconolactonase [Mus musculus]
7	7	0.101	protein disulfide isomerase associated 4 [Mus musculus]
2	2	0.0578	leucine aminopeptidase 3 [Mus musculus]

4.2 Proteins Identified by a Single Peptide

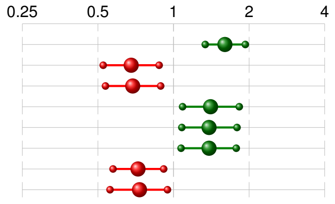

Peptides	Spectra	Coverage	Protein
1	11	0.068	hemoglobin Y, beta-like embryonic chain [Mus musculus]
1	1	NaN	PREDICTED: similar to Versican core protein precursor (Large fibroblast proteoglycan) (Chondroitin sulfate proteog
1	1	0.213	ATP synthase, H+ transporting, mitochondrial F0 complex, subunit F [Mus musculus]
1	1	0.0284	eukaryotic translation initiation factor 3, subunit 3 (gamma) [Mus musculus]
1	1	0.00763	PREDICTED: desmoplakin isoform 1 [Mus musculus]
1	1	0.0345	enoyl Coenzyme A hydratase, short chain, 1, mitochondrial [Mus musculus]
1	2	NaN	hypothetical protein LOC442796 [Mus musculus]
1	1	0.0541	protein kinase, cAMP dependent, catalytic, alpha [Mus musculus]

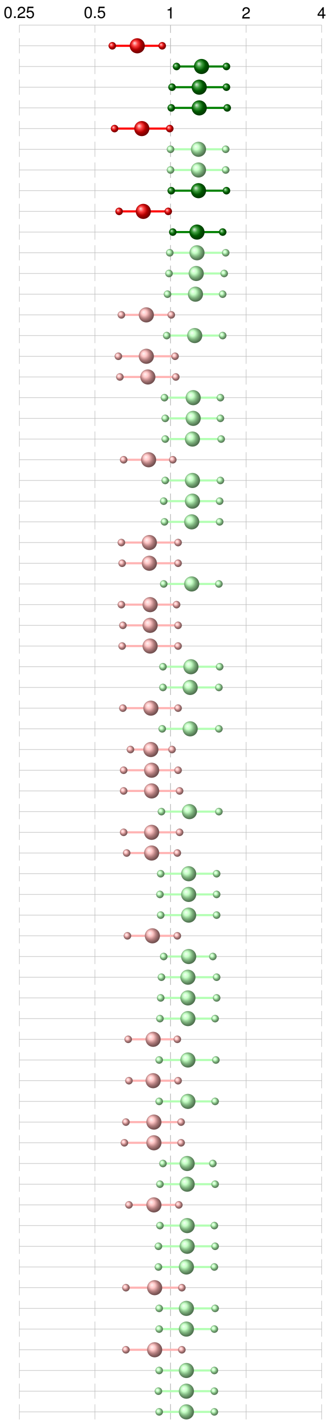

Peptides	Spectra	Coverage	Protein
1	2	0.103	mago-nashi homolog, proliferation-associated [Mus musculus]
1	2	NaN	PREDICTED: similar to 3-phosphoglycerate dehydrogenase [Mus musculus]
1	1	0.0456	microfibrillar-associated protein 1 [Mus musculus]
1	1	0.00838	UDP-glucose ceramide glucosyltransferase-like 1 [Mus musculus]
1	1	NaN	PREDICTED: similar to dynein, cytoplasmic, heavy polypeptide 2 [Mus musculus]
1	1	NaN	PREDICTED: similar to ribosomal protein L38 [Mus musculus]
1	1	0.0494	sorting nexin 3 [Mus musculus]
1	1	0.0185	dual specificity phosphatase 27 (putative) [Mus musculus]
1	2	0.202	ubiquinol-cytochrome c reductase hinge protein [Mus musculus]
1	2	0.0363	dynein, cytoplasmic, light intermediate chain 1 [Mus musculus]
1	1	0.0168	copine I [Mus musculus]
1	1	0.0467	TAF15 RNA polymerase II, TATA box binding protein (TBP)-associated factor [Mus musculus]
1	1	0.00669	nuclear mitotic apparatus protein 1 [Mus musculus]
1	2	0.0296	v-crk sarcoma virus CT10 oncogene homolog [Mus musculus]
1	1	0.0328	pigpen [Mus musculus]
1	1	NaN	nucleobindin 2 [Mus musculus]
1	1	0.0854	HD domain containing 2 [Mus musculus]
1	1	NaN	PREDICTED: similar to eukaryotic translation initiation factor 4A, isoform 1 isoform 1 [Mus musculus]
1	1	0.0223	hydroxyacyl-Coenzyme A dehydrogenase/3-ketoacyl-Coenzyme A thiolase/enoyl-Coenzyme A hydratase (trifunction
1	1	NaN	PREDICTED: similar to Histidine triad nucleotide-binding protein 2 (HINT-2) (HINT-3) [Mus musculus]
1	2	0.107	SH3-binding domain glutamic acid-rich protein [Mus musculus]
1	1	0.032	protease (prosome, macropain) 26S subunit, ATPase 5 [Mus musculus]
1	1	NaN	chaperonin subunit 2 (beta) [Mus musculus]
1	1	NaN	DEAD (Asp-Glu-Ala-Asp) box polypeptide 42 [Mus musculus]
1	1	NaN	PREDICTED: similar to Prothymosin alpha [Mus musculus]
1	1	0.0779	PREDICTED: similar to HESB like domain containing 1 isoform 5 [Mus musculus]
1	1	0.0147	hypothetical protein LOC227737 [Mus musculus]
1	1	0.0259	hypothetical protein LOC66622 [Mus musculus]
1	1	0.221	BolA-2 protein [Mus musculus]
1	1	NaN	PREDICTED: Purkinje cell protein 4-like 1 [Mus musculus]
1	1	0.0360	tubulin, beta 4 [Mus musculus]
1	1	0.034	karyopherin (importin) alpha 2 [Mus musculus]
1	1	0.162	limb-bud and heart [Mus musculus]
1	1	0.0358	galactokinase 2 [Mus musculus]
1	10	NaN	transmembrane protease, serine 13 [Mus musculus]
1	1	0.0393	Ymer protein isoform 1 [Mus musculus]
1	1	0.0358	protein phosphatase 4, catalytic subunit [Mus musculus]
1	1	0.0329	SH3-domain GRB2-like endophilin B2 [Mus musculus]
1	1	NaN	keratin complex 2, basic, gene 17 [Mus musculus]
1	2	0.110	RNA binding motif protein 8a [Mus musculus]
1	1	0.0251	mitogen activated protein kinase 1 [Mus musculus]
1	1	0.044	tubulin-specific chaperone c [Mus musculus]
1	1	0.037	cell division cycle 2 homolog A [Mus musculus]
1	2	NaN	PREDICTED: similar to craniofacial development protein 1 [Mus musculus]
1	2	0.0416	protein phosphatase-1 regulatory subunit 7 [Mus musculus]
1	1	0.0113	TBP-interacting protein b [Mus musculus]
1	1	0.0202	chaperonin subunit 3 (gamma) [Mus musculus]
1	1	NaN	insulin degrading enzyme [Mus musculus]
1	2	0.065	5’,3’-nucleotidase, cytosolic [Mus musculus]
1	1	0.0269	ATPase, H+ transporting, lysosomal, V1 subunit H [Mus musculus]
1	2	0.117	hematological and neurological expressed sequence 1 [Mus musculus]
1	1	0.067	PREDICTED: hypothetical protein LOC76020 [Mus musculus]
1	1	0.0340	caldesmon 1 [Mus musculus]
1	1	0.0265	annexin A2 [Mus musculus]
1	2	0.0270	tubulin, beta 2 [Mus musculus]
1	1	0.0264	proteasome 26S non-ATPase subunit 3 [Mus musculus]
1	2	NaN	PREDICTED: similar to 60S acidic ribosomal protein P2 isoform 2 [Mus musculus]
1	1	0.0435	ADP-ribosylation factor GTPase activating protein 1 [Mus musculus]
1	1	NaN	ubiquitin specific protease 9, X chromosome [Mus musculus]
1	1	0.0168	exportin 1, CRM1 homolog [Mus musculus]
1	1	NaN	PREDICTED: hypothetical protein [Mus musculus]
1	1	0.0153	ubiquitination factor E4B [Mus musculus]
1	1	0.0215	basic leucine zipper and W2 domains 2 [Mus musculus]
1	1	0.0535	peroxisome biogenesis factor 19 [Mus musculus]
1	1	0.0107	adaptor protein complex AP-2, alpha 2 subunit [Mus musculus]
1	1	0.0364	DnaJ (Hsp40) homolog, subfamily A, member 2 [Mus musculus]
1	1	0.00484	fibronectin 1 [Mus musculus]

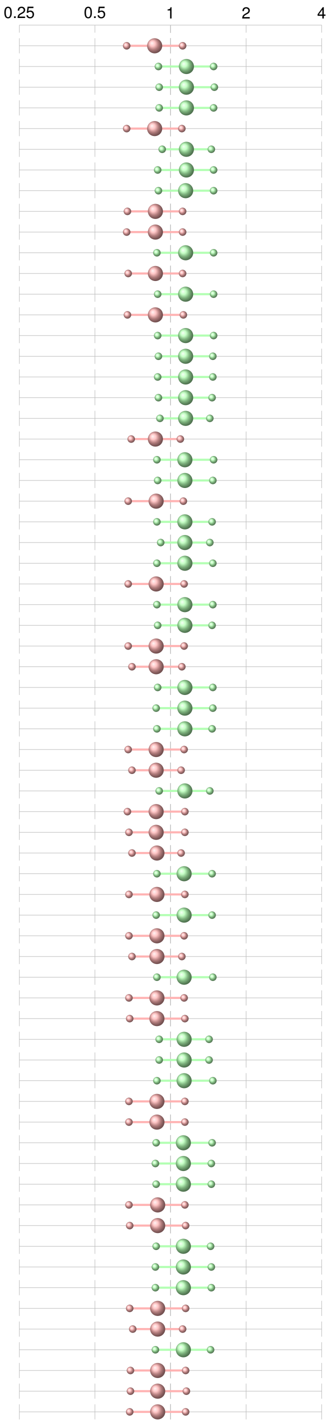

Peptides	Spectra	Coverage	Protein
1	1	0.0886	PREDICTED: similar to T-cell receptor alpha chain V region HPB-MLT precursor isoform 2 [Mus musculus]
1	1	0.03	aldo-keto reductase family 7, member A5 (aflatoxin aldehyde reductase) [Mus musculus]
1	1	0.0635	eukaryotic translation initiation factor 2, subunit 1 alpha [Mus musculus]
1	1	NaN	PREDICTED: similar to importin 11 isoform 3 [Mus musculus]
1	1	NaN	PREDICTED: similar to tumor protein, translationally-controlled 1 [Mus musculus]
1	2	NaN	poly(rC) binding protein 2 [Mus musculus]
1	1	0.0972	6-pyruvoyl-tetrahydropterin synthase [Mus musculus]
1	1	0.0268	ubiquilin 4 [Mus musculus]
1	1	0.0119	myosin VI [Mus musculus]
1	1	NaN	PREDICTED: similar to 14-3-3 protein theta (14-3-3 protein tau) [Mus musculus]
1	1	0.0371	sorting nexin 5 [Mus musculus]
1	1	0.055	ADP-ribosylation factor-like 3 [Mus musculus]
1	1	0.0201	protein tyrosine phosphatase, non-receptor type 11 [Mus musculus]
1	1	NaN	B-cell CLL/lymphoma 7B [Mus musculus]
1	1	0.0226	chaperonin subunit 6a (zeta) [Mus musculus]
1	1	NaN	hypothetical protein LOC433864 [Mus musculus]
1	1	0.0382	alveolar soft part sarcoma chromosome region, candidate 1 long isoform [Mus musculus]
1	1	0.0221	chaperonin subunit 7 (eta) [Mus musculus]
1	2	0.0389	cell division cycle 10 homolog [Mus musculus]
1	2	0.0438	C21orf19-like protein [Mus musculus]
1	1	0.0433	hypothetical protein LOC76820 [Mus musculus]
1	1	0.0259	succinate-Coenzyme A ligase, ADP-forming, beta subunit [Mus musculus]
1	1	0.0769	phosphoprotein enriched in astrocytes 15 isoform 2 [Mus musculus]
1	1	0.0250	aldehyde dehydrogenase 2, mitochondrial [Mus musculus]
1	2	0.0604	eukaryotic translation initiation factor 2, subunit 2 (beta) [Mus musculus]
1	1	0.0117	receptor tyrosine kinase-like orphan receptor 1 [Mus musculus]
1	1	NaN	cytochrome c oxidase, subunit Va [Mus musculus]
1	1	NaN	PREDICTED: similar to Succinyl-CoA ligase [GDP-forming] beta-chain, mitochondrial precursor (Succinyl-CoA synt
1	1	0.0347	asparaginyl-tRNA synthetase [Mus musculus]
1	1	0.0317	GGA binding partner [Mus musculus]
1	2	0.101	single-stranded DNA binding protein 1 isoform 1 [Mus musculus]
1	1	0.0174	minichromosome maintenance protein 4 [Mus musculus]
1	1	NaN	PREDICTED: similar to DnaJ-like protein 2 isoform 2 [Mus musculus]
1	1	NaN	ligase I, DNA, ATP-dependent [Mus musculus]
1	1	NaN	PREDICTED: similar to Diphosphoinositol polyphosphate phosphohydrolase 1 (DIPP-1) (muDIPP1) (Diadenosine 5,5
1	2	0.0112	reticulon 4 isoform A [Mus musculus]
1	2	0.00856	ubiquitin specific protease 28 [Mus musculus]
1	1	0.024	protein phosphatase 1G (formerly 2C), magnesium-dependent, gamma isoform [Mus musculus]
1	1	0.03	Rho, GDP dissociation inhibitor (GDI) beta [Mus musculus]
1	2	0.0833	ATP synthase, H+ transporting, mitochondrial F1 complex, delta subunit precursor [Mus musculus]
1	1	NaN	inter-alpha trypsin inhibitor, heavy chain 1 [Mus musculus]
1	1	0.0378	biphenyl hydrolase-like (serine hydrolase, breast epithelial mucin-associated antigen) [Mus musculus]
1	1	NaN	PREDICTED: similar to Myosin regulatory light chain 2, smooth muscle isoform (Myosin RLC) [Mus musculus]
1	1	0.0822	chromatin modifying protein 5 [Mus musculus]
1	2	0.0902	prefoldin 1 [Mus musculus]
1	1	NaN	HECT, UBA and WWE domain containing 1 [Mus musculus]
1	1	0.0319	KDEL (Lys-Asp-Glu-Leu) containing 1 [Mus musculus]
1	1	0.0629	Prpk (p53-related protein kinase)-binding protein [Mus musculus]
1	2	0.0483	cysteine and histidine-rich domain (CHORD)-containing, zinc-binding protein 1 [Mus musculus]
1	2	0.0324	peptidase (mitochondrial processing) alpha [Mus musculus]
1	1	0.0345	crystallin, zeta (quinone reductase)-like 1 [Mus musculus]
1	1	0.107	interferon alpha responsive [Mus musculus]
1	1	0.0289	ubiquilin 1 isoform 2 [Mus musculus]
1	1	0.0309	haloacid dehalogenase-like hydrolase domain containing 2 isoform 1 [Mus musculus]
1	1	0.0417	replication protein A1 [Mus musculus]
1	1	0.0173	3-oxoacid CoA transferase 1 [Mus musculus]
1	1	0.0256	villin 2 [Mus musculus]
1	1	0.0825	protein phosphatase 1, regulatory (inhibitor) subunit 1B [Mus musculus]
1	1	0.0203	EH domain containing protein MPAST2 [Mus musculus]
1	1	0.0346	ariadne homolog 2 [Mus musculus]
1	1	0.0299	nuclear receptor binding protein [Mus musculus]
1	1	0.0393	platelet-activating factor acetylhydrolase, isoform 1b, alpha2 subunit [Mus musculus]
1	2	0.0240	procollagen, type IV, alpha 3 (Goodpasture antigen) binding protein [Mus musculus]
1	1	NaN	COP9 signalosome subunit 8 [Mus musculus]
1	1	NaN	PREDICTED: similar to 5-methyltetrahydrofolate-homocysteine methyltransferase isoform 2 [Mus musculus]
1	1	0.0264	cysteine sulfinic acid decarboxylase [Mus musculus]
1	1	NaN	galactose-1-phosphate uridyl transferase [Mus musculus]

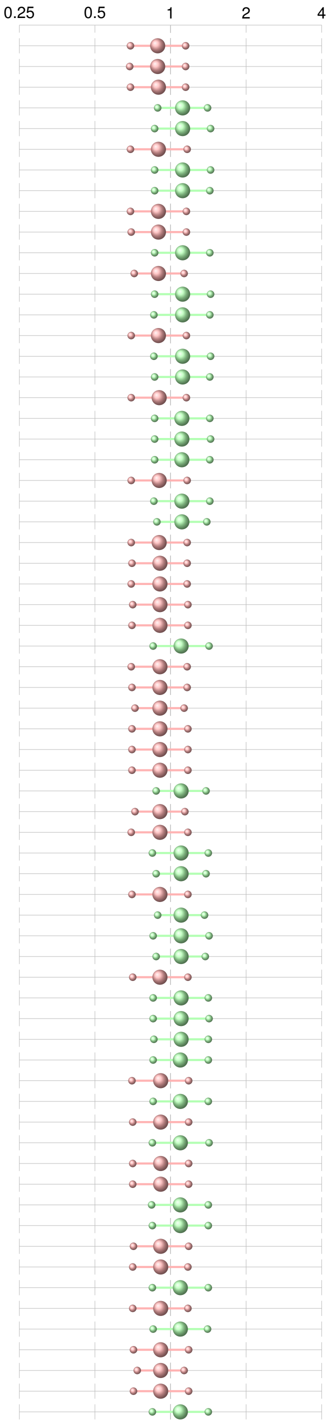

Peptides	Spectra	Coverage	Protein
1	1	0.0759	mesoderm development candiate 2 [Mus musculus]
1	1	0.0145	basal cell adhesion molecule [Mus musculus]
1	1	0.0492	protein phosphatase 6, catalytic subunit [Mus musculus]
1	2	0.0453	acetyl-Coenzyme A acyltransferase 2 (mitochondrial 3-oxoacyl-Coenzyme A thiolase) [Mus musculus]
1	1	0.0282	phosphoribosylaminoimidazole carboxylase [Mus musculus]
1	1	0.0884	small acidic protein [Mus musculus]
1	1	0.0208	glucosamine [Mus musculus]
1	1	NaN	leukotriene A4 hydrolase [Mus musculus]
1	1	NaN	keratin complex 2, basic, gene 8 [Mus musculus]
1	1	NaN	PREDICTED: similar to junctophilin 3 [Mus musculus]
1	1	0.0105	superkiller viralicidic activity 2-like [Mus musculus]
1	2	0.0747	male enhanced antigen 1 [Mus musculus]
1	1	0.0188	heterogenous nuclear ribonucleoprotein U [Mus musculus]
1	1	NaN	PREDICTED: similar to Ras-related protein Rab-11B isoform 3 [Mus musculus]
1	1	0.0172	HIV TAT specific factor 1 [Mus musculus]
1	1	0.0287	arsA (bacterial) arsenite transporter, ATP-binding, homolog 1 [Mus musculus]
1	1	0.0236	UDP-glucose pyrophosphorylase 2 [Mus musculus]
1	1	0.0337	methionyl aminopeptidase 1 [Mus musculus]
1	1	NaN	PREDICTED: hypothetical protein LOC76860 [Mus musculus]
1	1	0.075	huntingtin interacting protein 2 [Mus musculus]
1	1	0.0581	N-terminal Asn amidase [Mus musculus]
1	1	0.0302	heat shock protein, DNAJ-like 4 [Mus musculus]
1	1	0.0446	tropomodulin 1 [Mus musculus]
1	2	0.168	PREDICTED: similar to 60S acidic ribosomal protein P1 [Mus musculus]
1	1	0.0253	angio-associated migratory protein [Mus musculus]
1	1	0.091	PREDICTED: similar to Superoxide dismutase isoform 4 [Mus musculus]
1	1	0.0405	RNA binding motif, single stranded interacting protein 1 [Mus musculus]
1	1	0.0341	thioredoxin reductase 1 [Mus musculus]
1	1	0.0141	dihydrouridine synthase 3-like [Mus musculus]
1	1	0.063	superoxide dismutase 2, mitochondrial [Mus musculus]
1	1	0.0134	bicaudal D homolog 2 isoform 3 [Mus musculus]
1	1	NaN	tangerin [Mus musculus]
1	2	0.137	Tax1 (human T-cell leukemia virus type I) binding protein 3 [Mus musculus]
1	1	0.169	ATP synthase, H+ transporting, mitochondrial F1F0 complex, subunit e [Mus musculus]
1	1	NaN	SEC63-like [Mus musculus]
1	1	0.0242	type II keratin Kb37 [Mus musculus]
1	2	0.0581	myosin regulatory light chain-like [Mus musculus]
1	2	0.075	MARCKS-like protein [Mus musculus]
1	1	0.035	hypothetical protein LOC216618 [Mus musculus]
1	1	0.0633	PREDICTED: similar to Ubiquitin-conjugating enzyme E2 I (Ubiquitin-protein ligase I) (Ubiquitin carrier protein I) (SU
1	2	0.0160	asparagine synthetase [Mus musculus]
1	1	0.0323	paxillin isoform alpha [Mus musculus]
1	3	0.0202	splicing factor 3a, subunit 1 [Mus musculus]
1	1	0.0177	kinesin family member 5B [Mus musculus]
1	2	0.0247	ariadne ubiquitin-conjugating enzyme E2 binding protein homolog 1 [Mus musculus]
1	1	0.0462	cell-cycle related and expression-elevated protein in tumor [Mus musculus]
1	1	NaN	PREDICTED: alpha isoform of regulatory subunit B55, protein phosphatase 2 isoform 3 [Mus musculus]
1	1	0.0188	cytidine triphosphate synthase II [Mus musculus]
1	1	0.0299	O-sialoglycoprotein endopeptidase [Mus musculus]
1	1	0.0498	PREDICTED: similar to Proteasome subunit alpha type 5 (Proteasome zeta chain) (Macropain zeta chain) (Multicatal
1	1	0.0244	RAN binding protein 3 [Mus musculus]
1	1	0.00632	oxysterol binding protein-like 1 [Mus musculus]
1	1	0.0588	related RAS viral (r-ras) oncogene homolog 2 [Mus musculus]
1	1	0.0612	nudix (nucleoside diphosphate linked moiety X)-type motif 2 [Mus musculus]
1	1	0.118	LSM3 homolog, U6 small nuclear RNA associated [Mus musculus]
1	1	0.0203	solute carrier family 2 (facilitated glucose transporter), member 1 [Mus musculus]
1	1	0.00802	tight junction protein 1 [Mus musculus]
1	1	0.0264	ubiquitin specific protease 14 isoform 1 [Mus musculus]
1	1	0.0358	CNDP dipeptidase 2 (metallopeptidase M20 family) [Mus musculus]
1	1	0.0355	citrate lyase beta like [Mus musculus]
1	1	0.0299	golgi associated, gamma adaptin ear containing, ARF binding protein 2 [Mus musculus]
1	1	0.0559	PREDICTED: hypothetical protein [Mus musculus]
1	1	NaN	heat shock protein 105 [Mus musculus]
1	1	0.045	disintegrin metalloprotease (decysin) [Mus musculus]
1	3	0.00786	bromodomain containing 4 isoform 1 [Mus musculus]
1	1	0.167	hypothetical protein LOC624855 [Mus musculus]
1	1	NaN	PREDICTED: similar to Cullin-associated NEDD8-dissociated protein 1 (Cullin-associated and neddylation-dissociat

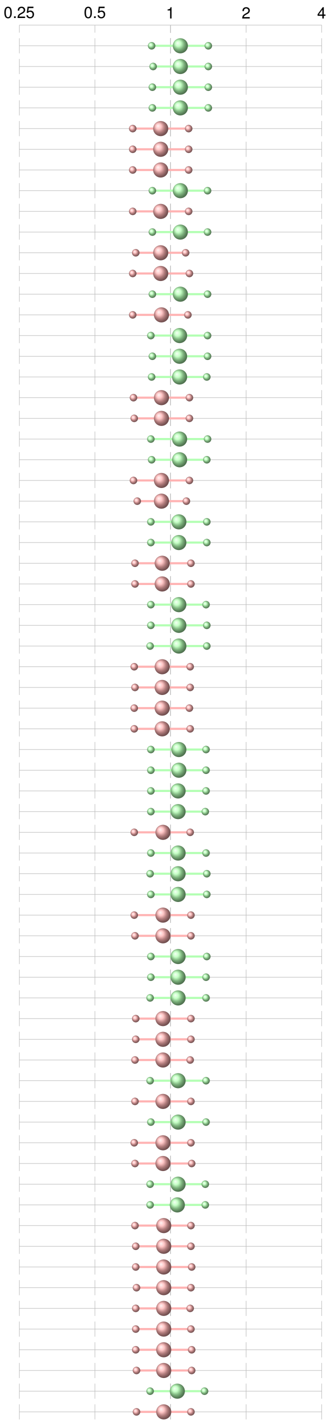

Peptides	Spectra	Coverage	Protein
1	1	0.0264	UDP-glucose dehydrogenase [Mus musculus]
1	1	0.0493	4-nitrophenylphosphatase domain and non-neuronal SNAP25-like protein homolog 1 [Mus musculus]
1	1	0.0108	phospholipase C, gamma 1 [Mus musculus]
1	1	NaN	FYVE and coiled-coil domain containing 1 [Mus musculus]
1	1	NaN	laminin B1 subunit 1 [Mus musculus]
1	1	NaN	PREDICTED: similar to Guanine nucleotide-binding protein G(i), alpha-2 subunit (Adenylate cyclase-inhibiting G alp
1	1	0.0882	ubiquitin-conjugating enzyme E2G 1 [Mus musculus]
1	1	0.0243	peptidylprolyl isomerase D [Mus musculus]
1	1	0.119	C10 protein [Mus musculus]
1	1	NaN	PREDICTED: similar to Kelch-like protein 3 [Mus musculus]
1	2	NaN	PREDICTED: similar to PEST-containing nuclear protein (PCNP) [Mus musculus]
1	1	0.0286	bleomycin hydrolase [Mus musculus]
1	1	0.0312	apolipoprotein B editing complex 2 [Mus musculus]
1	1	0.0158	FK506 binding protein 9 [Mus musculus]
1	1	0.0179	cullin 4A [Mus musculus]
1	1	0.0149	acetoacetyl-CoA synthetase [Mus musculus]
1	1	NaN	gelsolin-like capping protein [Mus musculus]
1	1	0.0583	cytokine induced apoptosis inhibitor 1 [Mus musculus]
1	1	0.156	high mobility group nucleosomal binding domain 1 [Mus musculus]
1	1	NaN	PREDICTED: similar to H2A histone family, member O [Mus musculus]
1	1	0.0472	phosphate cytidylyltransferase 1, choline, beta isoform isoform 2 [Mus musculus]
1	1	0.0498	transcription elongation factor A (SII) 1 [Mus musculus]
1	2	0.0166	SAPS domain family, member 3 [Mus musculus]
1	1	0.0173	aldehyde dehydrogenase 1 family, member L2 [Mus musculus]
1	1	0.0331	adaptor-related protein complex AP-1, mu subunit 1 [Mus musculus]
1	1	0.114	thioredoxin-like 5 [Mus musculus]
1	1	0.158	PREDICTED: similar to SMT3 suppressor of mif two 3 homolog 2 [Mus musculus]
1	1	0.013	matrin 3 [Mus musculus]
1	1	0.00716	ubiquitin-activating enzyme E1-like [Mus musculus]
1	1	0.0130	brain glycogen phosphorylase [Mus musculus]
1	1	0.0476	NAD(P)H dehydrogenase, quinone 2 [Mus musculus]
1	1	0.0472	low density lipoprotein receptor-related protein associated protein 1 [Mus musculus]
1	1	0.110	PREDICTED: similar to Nuclear transport factor 2 (NTF-2) [Mus musculus]
1	1	0.0671	hypothetical protein LOC67914 [Mus musculus]
1	1	0.141	acylphosphatase 1, erythrocyte (common) type [Mus musculus]
1	1	NaN	NIMA-related expressed kinase 9 [Mus musculus]
1	1	NaN	heterogeneous nuclear ribonucleoprotein D-like [Mus musculus]
1	1	0.0335	Ddx19-like protein [Mus musculus]
1	1	0.0303	drebrin 1 [Mus musculus]
1	1	0.0262	procollagen-proline, 2-oxoglutarate 4-dioxygenase (proline 4-hydroxylase), alpha 1 polypeptide [Mus musculus]
1	1	NaN	PREDICTED: similar to FUS-interacting serine-arginine-rich protein 1 (TLS-associated protein with Ser-Arg repeats)
1	1	0.0183	dynamin 1-like isoform a [Mus musculus]
1	1	NaN	S-phase kinase-associated protein 1A [Mus musculus]
1	1	0.04	preimplantation protein 3 [Mus musculus]
1	1	0.0481	hypothetical protein LOC76429 [Mus musculus]
1	1	0.0791	divalent cation tolerant protein CUTA isoform 1 [Mus musculus]
1	1	0.0411	tRNA splicing endonuclease 34 [Mus musculus]
1	1	0.0307	kinesin-like 8 [Mus musculus]
1	1	0.0261	protein phosphatase 5, catalytic subunit [Mus musculus]
1	1	0.156	prefoldin 4 [Mus musculus]
1	1	NaN	hypothetical protein LOC227712 [Mus musculus]
1	1	0.0488	PKC-potentiated PP1 inhibitory protein [Mus musculus]
1	1	NaN	hypothetical protein LOC71970 [Mus musculus]
1	1	0.00695	suppressor of Ty 6 homolog [Mus musculus]
1	1	0.091	protein (peptidyl-prolyl cis/trans isomerase) NIMA-interacting 1 [Mus musculus]
1	1	0.0561	N6-DNA methyltransferase A [Mus musculus]
1	1	0.0202	signal transducer and activator of transcription 5A [Mus musculus]
1	1	0.0802	heat shock protein, alpha-crystallin-related, B6 [Mus musculus]
1	1	0.0856	dihydrofolate reductase [Mus musculus]
1	1	NaN	PREDICTED: NADH dehydrogenase (ubiquinone) flavoprotein 2 isoform 3 [Mus musculus]
1	1	0.0859	guanylate kinase 1 [Mus musculus]
1	1	NaN	RIKEN cDNA G430041M01 [Mus musculus]
1	1	0.113	polyadenylate-binding protein-interacting protein 2 [Mus musculus]
1	1	0.0603	histone cell cycle regulation defective interacting protein 5 [Mus musculus]
1	1	0.0326	BRG1/brm-associated factor 53A [Mus musculus]
1	1	0.0185	growth factor receptor bound protein 10 [Mus musculus]
1	1	0.0364	poly(A) binding protein, nuclear 1 [Mus musculus]

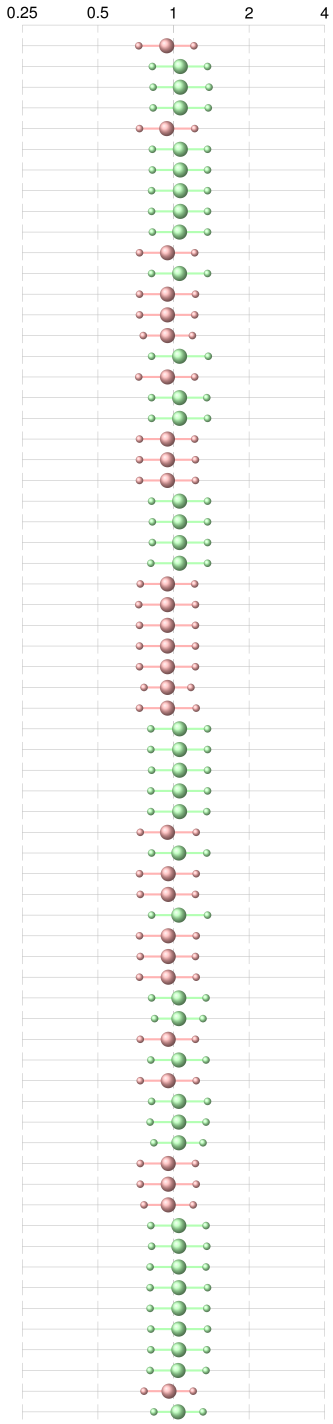

Peptides	Spectra	Coverage	Protein
1	1	NaN	utrophin [Mus musculus]
1	1	0.0512	casein kinase II, beta subunit [Mus musculus]
1	1	0.114	calmodulin 2 [Mus musculus]
1	1	NaN	PREDICTED: similar to CG1814-PA, isoform A [Mus musculus]
1	1	NaN	PREDICTED: similar to multi sex combs CG12058-PA [Mus musculus]
1	1	0.0431	3-hydroxy-3-methylglutaryl-Coenzyme A lyase [Mus musculus]
1	1	NaN	eukaryotic translation termination factor 1 [Mus musculus]
1	1	0.0206	pleckstrin homology domain containing, family C (with FERM domain) member 1 [Mus musculus]
1	1	0.0218	phosphofructokinase, liver, B-type [Mus musculus]
1	1	0.0239	ARP3 actin-related protein 3 homolog [Mus musculus]
1	1	0.0283	HIRA interacting protein 3 [Mus musculus]
1	1	0.0111	minichromosome maintenance deficient 3 [Mus musculus]
1	1	0.0478	PREDICTED: similar to Nidogen-1 precursor (Entactin) [Mus musculus]
1	1	0.0134	nucleoporin 93 [Mus musculus]
1	2	0.0233	protein O-fucosyltransferase 2 [Mus musculus]
1	1	0.0221	protein phosphatase 1F (PP2C domain containing) [Mus musculus]
1	1	0.0536	sepiapterin reductase [Mus musculus]
1	1	NaN	adducin 1 (alpha) isoform 1 [Mus musculus]
1	1	0.0566	histone 1, H1c [Mus musculus]
1	1	0.0945	H2-K region expressed gene 2 [Mus musculus]
1	1	0.0425	solute carrier family 7, member 6 opposite strand [Mus musculus]
1	1	0.0632	chloride intracellular channel 4 (mitochondrial) [Mus musculus]
1	1	0.163	enhancer of rudimentary homolog [Mus musculus]
1	1	0.0796	RAB1B, member RAS oncogene family [Mus musculus]
1	1	0.0377	phosphoribosyl pyrophosphate synthetase 1-like 1 [Mus musculus]
1	1	0.0289	caspase 3, apoptosis related cysteine protease [Mus musculus]
1	1	0.1	profilin 2 [Mus musculus]
1	1	0.0538	associated with Prkcl1 [Mus musculus]
1	1	0.0267	aldehyde dehydrogenase 4 family, member A1 [Mus musculus]
1	1	0.0763	transcription elongation factor B (SIII), polypeptide 2 [Mus musculus]
1	1	0.0487	selenium binding protein 1 [Mus musculus]
1	3	0.0383	cathepsin B preproprotein [Mus musculus]
1	1	NaN	PREDICTED: similar to hydroxymethylbilane synthase [Mus musculus]
1	1	0.00811	STE20-like kinase [Mus musculus]
1	1	0.0207	zinc finger protein 326 [Mus musculus]
1	1	NaN	PREDICTED: similar to discs large homolog 1 isoform 3 [Mus musculus]
1	1	NaN	PREDICTED: similar to G protein pathway suppressor 1 isoform 2 [Mus musculus]
1	1	0.041	hypothetical protein LOC68045 [Mus musculus]
1	1	0.0588	glycine cleavage system protein H (aminomethyl carrier) [Mus musculus]
1	1	0.0261	calcium/calmodulin-dependent protein kinase II, delta isoform 2 [Mus musculus]
1	1	0.0608	ADP-ribosylation factor 1 [Mus musculus]
1	1	0.0573	sperm associated antigen 7 [Mus musculus]
1	1	0.0369	eukaryotic translation initiation factor 3, subunit 2 (beta) [Mus musculus]
1	1	NaN	PREDICTED: similar to protein phosphatase 4, regulatory subunit 2 [Mus musculus]
1	1	0.0583	glutathione S-transferase omega 1 [Mus musculus]
1	1	0.0396	lymphocyte specific 1 [Mus musculus]
1	1	NaN	thymopoietin [Mus musculus]
1	2	0.0648	acidic (leucine-rich) nuclear phosphoprotein 32 family, member A [Mus musculus]
1	1	0.0313	ubiquilin 2 [Mus musculus]
1	1	0.0156	procollagen, type VI, alpha 1 [Mus musculus]
1	1	0.0325	histidine triad protein member 5 [Mus musculus]
1	1	0.0431	actin-related protein 2 [Mus musculus]
1	1	NaN	PREDICTED: amylo-1,6-glucosidase, 4-alpha-glucanotransferase isoform 2 [Mus musculus]
1	2	0.0364	calponin 3, acidic [Mus musculus]
1	1	0.129	protein phosphatase 1, regulatory (inhibitor) subunit 1A [Mus musculus]
1	1	NaN	RAB7, member RAS oncogene family [Mus musculus]
1	2	NaN	PREDICTED: similar to protein phosphatase 1, regulatory (inhibitor) subunit 14B [Mus musculus]
1	1	0.0257	RAB3A interacting protein [Mus musculus]
1	1	NaN	potassium channel tetramerisation domain containing 12 [Mus musculus]
1	1	0.0203	transforming growth factor beta 1 induced transcript 1 [Mus musculus]
1	1	0.0175	aldehyde dehydrogenase 16 family, member A1 [Mus musculus]
1	1	0.0308	AMP-activated protein kinase alpha 2 catalytic subunit [Mus musculus]
1	1	0.0369	coxsackievirus and adenovirus receptor isoform b [Mus musculus]
1	1	NaN	PREDICTED: similar to Microtubule-actin crosslinking factor 1 (Actin cross-linking family 7) isoform 2 [Mus musculu
1	1	0.02	tubulin, beta 3 [Mus musculus]
1	2	NaN	PREDICTED: similar to vacuolar H+ ATPase G1 [Mus musculus]
1	2	0.0586	glutathione S-transferase, alpha 4 [Mus musculus]

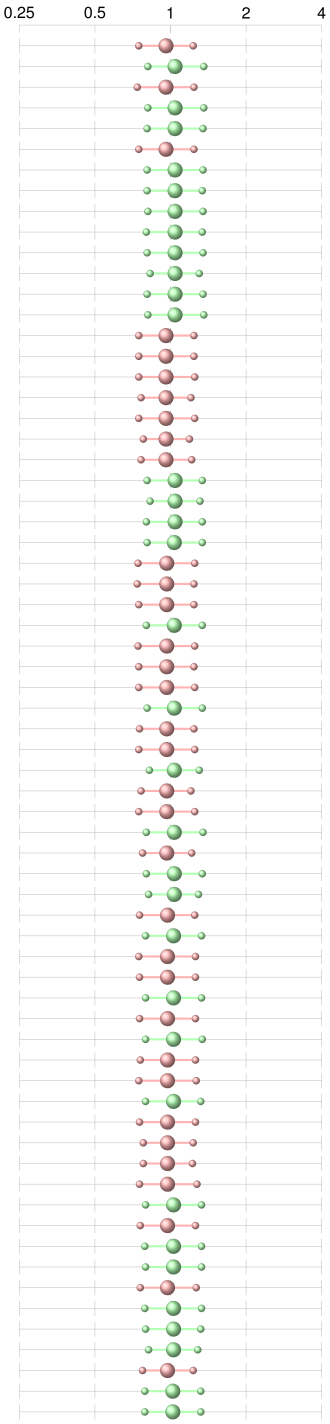

Peptides	Spectra	Coverage	Protein
1	1	0.0427	protein phosphatase 1, regulatory (inhibitor) subunit 8 [Mus musculus]
1	1	0.0265	serine (or cysteine) proteinase inhibitor, clade B, member 6a [Mus musculus]
1	1	0.0223	exocyst complex component 8 [Mus musculus]
1	1	0.0279	fatso [Mus musculus]
1	1	0.0180	myo-inositol 1-phosphate synthase A1 [Mus musculus]
1	1	0.043	alpha 1 microglobulin/bikunin [Mus musculus]
1	1	0.0178	histidyl-tRNA synthetase-like [Mus musculus]
1	1	0.0264	dihydrolipoamide S-succinyltransferase (E2 component of 2-oxo-glutarate complex) [Mus musculus]
1	1	0.0421	proteasome (prosome, macropain) subunit, alpha type 4 [Mus musculus]
1	1	0.112	selenoprotein H [Mus musculus]
1	1	NaN	kinesin family member 13A [Mus musculus]
1	2	0.0677	Bcl2-associated X protein [Mus musculus]
1	1	0.00816	PREDICTED: similar to Bifunctional aminoacyl-tRNA synthetase [Mus musculus]
1	1	0.0442	endonuclease G [Mus musculus]
1	1	NaN	von Hippel-Lindau binding protein 1 [Mus musculus]
1	1	0.019	procollagen lysine, 2-oxoglutarate 5-dioxygenase 2 [Mus musculus]
1	1	0.0395	STIP1 homology and U-box containing protein 1 [Mus musculus]
1	2	0.0563	coactosin-like 1 [Mus musculus]
1	1	0.0289	c-src tyrosine kinase [Mus musculus]
1	3	0.081	trypsinogen 7 [Mus musculus]
1	2	0.0433	PREDICTED: similar to Carbonyl reductase [NADPH] 1 (NADPH-dependent carbonyl reductase 1) [Mus musculus]
1	1	NaN	PREDICTED: similar to C-terminal-binding protein 1 (CtBP1) (C-terminal-binding protein 3) (CtBP3) (50 kDa BFA-dep
1	2	0.0149	seryl-aminoacyl-tRNA synthetase 1 [Mus musculus]
1	1	0.0563	family with sequence similarity 96, member A [Mus musculus]
1	1	0.0143	heat shock 70kDa protein 4 like [Mus musculus]
1	1	0.00888	procollagen, type III, alpha 1 [Mus musculus]
1	1	0.0175	chromosome segregation 1-like [Mus musculus]
1	1	0.0511	toll interacting protein [Mus musculus]
1	1	0.0333	PREDICTED: similar to interferon regulatory factor 2 binding protein 2 isoform 1 [Mus musculus]
1	1	NaN	minichromosome maintenance deficient 5, cell division cycle 46 [Mus musculus]
1	1	0.0362	N-acetylneuraminic acid synthase (sialic acid synthase) [Mus musculus]
1	1	0.0778	Ufm1-conjugating enzyme 1 [Mus musculus]
1	1	0.0169	Fas-associated factor 1 [Mus musculus]
1	1	0.0113	early endosome antigen 1 [Mus musculus]
1	1	NaN	PREDICTED: similar to phosphatase and actin regulator 4 [Mus musculus]
1	2	0.0648	solute carrier family 9 (sodium/hydrogen exchanger), isoform 3 regulator 1 [Mus musculus]
1	2	0.167	U6 snRNA-associated Sm-like protein LSm8 [Mus musculus]
1	1	0.0197	hypothetical protein LOC94184 isoform 3 [Mus musculus]
1	1	0.0552	epsilon subunit of coatomer protein complex [Mus musculus]
1	2	NaN	PREDICTED: similar to deoxyuridine triphosphatase [Mus musculus]
1	1	0.167	PREDICTED: similar to Nonhistone chromosomal protein HMG-17 (High-mobility group nucleosome binding domain
1	2	0.0794	small nuclear ribonucleoprotein D3 [Mus musculus]
1	1	0.0448	dynactin 2 [Mus musculus]
1	1	0.0497	ADP-ribosylarginine hydrolase [Mus musculus]
1	1	0.0359	mitochondrial ribosomal protein L45 [Mus musculus]
1	1	0.0495	splicing factor, arginine/serine rich 9 [Mus musculus]
1	1	0.0122	coatomer protein complex, subunit beta 2 (beta prime) [Mus musculus]
1	1	NaN	PREDICTED: hypothetical protein [Mus musculus]
1	1	0.0399	zinc binding alcohol dehydrogenase, domain containing 1 [Mus musculus]
1	1	0.107	c-myc binding protein [Mus musculus]
1	1	0.0478	3-hydroxyisobutyrate dehydrogenase, mitochondrial precursor [Mus musculus]
1	1	0.0296	thioredoxin domain containing 4 [Mus musculus]
1	1	0.108	calcium-regulated heat-stable protein (24kD) [Mus musculus]
1	2	NaN	PREDICTED: similar to Protein farnesyltransferase/geranylgeranyltransferase type I alpha subunit (CAAX farnesyltra
1	2	0.0295	muscleblind-like 2 isoform 1 [Mus musculus]
1	1	NaN	fibulin 2 [Mus musculus]
1	1	NaN	RAP1, GTP-GDP dissociation stimulator 1 [Mus musculus]
1	1	0.0292	inhibitor of kappaB kinase gamma isoform 2 [Mus musculus]
1	1	NaN	hypothetical protein LOC68646 [Mus musculus]
1	1	0.0161	N-ethylmaleimide sensitive fusion protein [Mus musculus]
1	1	0.00318	AHNAK nucleoprotein isoform 1 [Mus musculus]
1	1	0.019	heterogeneous nuclear ribonucleoprotein R [Mus musculus]
1	1	0.0308	RAD9 homolog [Mus musculus]
1	2	0.0276	legumain [Mus musculus]
1	2	0.0383	splicing factor, arginine/serine-rich 4 (SRp75) [Mus musculus]
1	1	0.0287	cytoskeleton-associated protein 1 [Mus musculus]
1	1	NaN	protease, serine, 15 [Mus musculus]

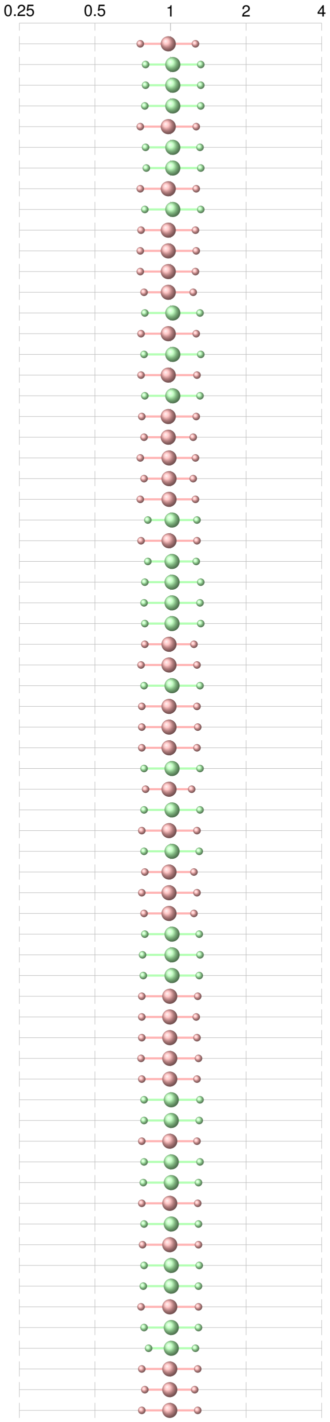

Peptides	Spectra	Coverage	Protein
1	1	NaN	PREDICTED: similar to Diphosphoinositol polyphosphate phosphohydrolase 2 (DIPP-2) (Diadenosine 5,5-P1,P6-hex
1	1	NaN	PREDICTED: similar to odd Oz/ten-m homolog 2 [Mus musculus]
1	1	0.0391	ubiquitin fusion degradation 1 like [Mus musculus]
1	1	NaN	PREDICTED: similar to OTU domain-containing protein 3 [Mus musculus]
1	1	0.0301	LanC (bacterial lantibiotic synthetase component C)-like 1 [Mus musculus]
1	1	NaN	PREDICTED: similar to Protein C9orf64 [Mus musculus]
1	1	0.0212	phosphoglucomutase 5 [Mus musculus]
1	1	0.0296	COP9 signalosome subunit 4 [Mus musculus]
1	1	0.0775	glia maturation factor, beta [Mus musculus]
1	1	0.00917	ubiquitin specific protease 15 [Mus musculus]
1	1	0.0205	karyopherin (importin) alpha 6 [Mus musculus]
1	1	0.0672	microtubule-associated protein, RP/EB family, member 1 [Mus musculus]
1	2	NaN	hypothetical protein LOC432987 [Mus musculus]
1	1	0.0506	basic transcription factor 3-like 4 [Mus musculus]
1	1	0.0122	ribosomal protein S6 kinase polypeptide 3 [Mus musculus]
1	1	0.0306	Tial1 cytotoxic granule-associated RNA binding protein-like 1 [Mus musculus]
1	1	0.00781	PREDICTED: similar to ADAMTS-like 3 [Mus musculus]
1	1	0.0311	eukaryotic translation elongation factor 1 beta 2 [Mus musculus]
1	1	0.0183	zinc finger, CCHC domain containing 8 [Mus musculus]
1	2	0.0152	liver-specific bHLH-Zip transcription factor [Mus musculus]
1	1	0.0143	papillary renal cell carcinoma translocation-associated gene product [Mus musculus]
1	2	0.0187	lamin B1 [Mus musculus]
1	1	0.0170	G1 to S phase transition 1 [Mus musculus]
1	2	NaN	uroporphyrinogen decarboxylase [Mus musculus]
1	1	0.0333	zinc finger protein 265 [Mus musculus]
1	2	NaN	leucine-rich PPR motif-containing protein [Mus musculus]
1	1	0.0389	methionine adenosyltransferase II, beta [Mus musculus]
1	1	0.0369	phosphatidylinositol transfer protein, beta [Mus musculus]
1	1	0.0791	PREDICTED: similar to peptidylprolyl isomerase H [Mus musculus]
1	2	0.0638	guanosine monophosphate reductase [Mus musculus]
1	1	0.0357	FK506 binding protein 3 [Mus musculus]
1	1	0.0120	Sec23 interacting protein [Mus musculus]
1	1	NaN	PREDICTED: similar to p130Cas-associated protein (p140Cap) (SNAP-25-interacting protein) (SNIP) isoform 4 [Mus r
1	1	0.016	A kinase anchor protein 8 [Mus musculus]
1	1	0.118	PHF5A [Mus musculus]
1	1	0.0438	peroxiredoxin 4 [Mus musculus]
1	3	NaN	PREDICTED: similar to protease, serine, 3 [Mus musculus]
1	1	NaN	retinoblastoma binding protein 7 [Mus musculus]
1	1	0.0429	cyclin-dependent kinase 4 [Mus musculus]
1	1	0.0651	RAB14, member RAS oncogene family [Mus musculus]
1	2	NaN	PREDICTED: similar to cell division cycle 34 homolog isoform 2 [Mus musculus]
1	1	0.0855	tetratricopeptide repeat domain 11 [Mus musculus]
1	2	0.0386	eukaryotic translation initiation factor 4B [Mus musculus]
1	1	0.0312	tropomodulin 3 [Mus musculus]
1	1	NaN	coronin 7 [Mus musculus]
1	1	NaN	PREDICTED: similar to pyruvate dehydrogenase (lipoamide) beta [Mus musculus]
1	1	0.0185	melanoma cell adhesion molecule [Mus musculus]
1	1	NaN	PREDICTED: hypothetical protein LOC73713 [Mus musculus]
1	1	0.0609	hypothetical protein LOC223978 [Mus musculus]
1	1	NaN	PREDICTED: similar to Gamma-soluble NSF attachment protein (SNAP-gamma) (N-ethylmaleimide-sensitive factor a
1	1	NaN	PREDICTED: similar to TSC22 domain family protein 2 (TSC22-related-inducible leucine zipper protein 4) [Mus musc
1	1	NaN	PREDICTED: similar to nudix (nucleoside diphosphate linked moiety X)-type motif 9 [Mus musculus]
1	1	0.0209	SEC23A [Mus musculus]
1	1	0.0297	phosphoserine aminotransferase 1 [Mus musculus]
1	1	0.0417	myozenin 2 [Mus musculus]
1	1	0.00719	restin [Mus musculus]
1	1	0.0200	nucleolar and coiled-body phosphoprotein 1 isoform D [Mus musculus]
1	1	NaN	PREDICTED: similar to Splicing factor, arginine/serine-rich 5 (Pre-mRNA splicing factor SRP40) (Insulin-induced gro
1	1	0.0137	RAN binding protein 5 [Mus musculus]
1	1	NaN	cleavage stimulation factor, 3' pre-RNA subunit 2, tau [Mus musculus]
1	1	0.0423	RNA binding motif, single stranded interacting protein 2 isoform b [Mus musculus]
1	1	NaN	CUG triplet repeat, RNA binding protein 2 [Mus musculus]
1	1	NaN	PREDICTED: similar to microtubule-associated protein 1 A isoform 5 [Mus musculus]
1	3	0.0818	steroid receptor RNA activator 1 [Mus musculus]
1	1	0.0249	histone deacetylase 1 [Mus musculus]
1	2	0.037	peptidylprolyl isomerase B [Mus musculus]
1	1	0.0395	DAZ associated protein 1 [Mus musculus]
