# Supplementary material for: Imbalanced Expression of Vcan mRNA Splice Form Proteins Alters Heart Morphology and Cellular Protein Profiles
Source: PLoS One. 2014 Feb 20;9(2):e89133. doi: 10.1371/journal.pone.0089133 (PMC3930639; doi:10.1371/journal.pone.0089133)
Supplement: File S2 — Supplemental GO file. (PDF) [file pone.0089133.s002.pdf]

TABLE 1.Proteins within selected gene ontology (GO) categories that likely underlie the observed null heart and vasculature phenotype.

GO Identifier	GO Term Description	Count	Differentially Expressed Gene Names
GO:0006936	muscle contraction	7	Tpm1 Myom1 Tpm4 Myh7 Tpm3 Fkbp1a Tln1
GO:0007275	multicellular organismal development	5	Nes Tpi1 Csrp3 Stmn1 Dpysl2
GO:0001701	in utero embryonic development	5	Myh10 Tpm1 Ybx1 Srsf1 Hba-a1
GO:0008283	cell proliferation	5	Myh10 Prdx1 Rac1 Nasp Park7
GO:0055010	ventricular cardiac muscle tissue morphogenesis	5	Tpm1 Myl3 Myl2 Myh7 Tnnc1
GO:0060048	cardiac muscle contraction	5	Tpm1 Myl3 Myl4 Tnnc1 Srsf1
GO:0006915	apoptosis	5	Pdcd5 Rac1 Bag3 Eif5a Ywhae
GO:0007155	cell adhesion	5	Rac1 **Vcan** Fbln5 Rhoa Tln1
GO:0043066	negative regulation of apoptosis	5	Bag3 Usp47 Ybx1 Eif5a Alb
GO:0007399	nervous system development	4	Nes Dpysl3 Stmn1 Dpysl2
GO:0008284	positive regulation of cell proliferation	4	Ybx1 Calr Eif5a Crip2
GO:0002026	regulation of the force of heart contraction	4	Myl3 Myl4 Csrp3 Pebp1
GO:0007165	signal transduction	4	Arhgap1 Akap12 Ywhaq Fkbp1a
GO:0030154	cell differentiation	4	Csrp3 Stmn1 Dpysl2 Rhoa
GO:0009790	embryo development	3	Tpm1 Tpi1 Atp5a1
GO:0001525	angiogenesis	3	Ncl Atp5b Gpi1
GO:0007507	heart development	3	Mb Myl2 **Vcan**
GO:0000902	cell morphogenesis	3	Cap1 Cap2 Rhoa
GO:0007067	mitosis	3	Dync1li1 Mapre2 Cltc
GO:0051301	cell division	3	Dync1li1 Mapre2 Cdc42
GO:0030334	regulation of cell migration	3	Rac1 Nexn Tmsb4x
GO:0043524	negative regulation of neuron apoptosis	3	Set Gpi1 Rhoa
GO:0007512	adult heart development	2	Myh10 Myh7
GO:0008360	regulation of cell shape	2	Myh10 Aldoa
GO:0055003	cardiac myofibril assembly	2	Myh10 Myl2
GO:0008624	induction of apoptosis by extracellular signals	2	Rac1 Ywhae
GO:0016477	cell migration	2	Rac1 Fscn1
GO:0042981	regulation of apoptosis	2	Bag3 Actn4
GO:0030307	positive regulation of cell growth	2	Usp47 Rhoa
GO:0007517	muscle organ development	2	Des Csrp3
GO:0045666	positive regulation of neuron differentiation	2	Mtap1b Rhoa
GO:0016337	cell-cell adhesion	2	Dsp Cdc42
GO:0043588	skin development	2	Dsp Dbi

GO:0045665	negative regulation of neuron differentiation	2	Calr Rhoa
GO:0051149	positive regulation of muscle cell differentiation	2	Eif5a Cdc42
GO:0045664	regulation of neuron differentiation	2	Ywhag Dpysl2
GO:0050770	regulation of axonogenesis	2	Rhoa Arhgdia
GO:0050771	negative regulation of axonogenesis	2	Rhoa Arhgdia
GO:0050772	positive regulation of axonogenesis	2	Rhoa Arhgdia
GO:0043065	positive regulation of apoptosis	1	Anxa5
GO:0048858	cell projection morphogenesis	1	Nes
GO:2000179	positive regulation of neural precursor cell proliferation	1	Nes
GO:0055015	ventricular cardiac muscle cell development	1	Myh10
GO:0045214	sarcomere organization	1	Tpm1
GO:0060047	heart contraction	1	Myl2
GO:0012501	programmed cell death	1	Pkm2
GO:0010862	positive regulation of pathway-restricted SMAD protein phosphorylation	1	Rbpms
GO:0060391	positive regulation of SMAD protein import into nucleus	1	Rbpms
GO:0001824	blastocyst development	1	Nasp
GO:0002027	regulation of heart rate	1	Myh7
GO:0007050	cell cycle arrest	1	Calr
GO:0040020	regulation of meiosis	1	Calr
GO:0045787	positive regulation of cell cycle	1	Calr
GO:0048387	negative regulation of retinoic acid receptor signaling pathway	1	Calr
GO:0090398	cellular senescence	1	Calr
GO:0006917	induction of apoptosis	1	Eif5a
GO:0030199	collagen fibril organization	1	Serpinh1
GO:0032964	collagen biosynthetic process	1	Serpinh1
GO:0060395	SMAD protein signal transduction	1	Afp
GO:0007183	SMAD protein complex assembly	1	Fkbp1a
GO:0030198	extracellular matrix organization	1	Fbln5
GO:0048251	elastic fiber assembly	1	Fbln5
GO:0008016	regulation of heart contraction	1	Hrc
GO:0060684	epithelial-mesenchymal cell signaling	1	Cdc42
GO:0007160	cell-matrix adhesion	1	Rhoa
GO:0007229	integrin-mediated signaling pathway	1	Rhoa
